# Protein–protein interactions between tenascin-R and RPTPζ/phosphacan are critical to maintain the architecture of perineuronal nets

**DOI:** 10.1016/j.jbc.2023.104952

**Published:** 2023-06-23

**Authors:** Ashis Sinha, Jessica Kawakami, Kimberly S. Cole, Aliona Ladutska, Mary Y. Nguyen, Mary S. Zalmai, Brandon L. Holder, Victor M. Broerman, Russell T. Matthews, Samuel Bouyain

**Affiliations:** 1 Department of Neuroscience and Physiology, State University of New York Upstate Medical University, Syracuse, New York, USA; 2 Division of Biological and Biomedical Systems, School of Science and Engineering, University of Missouri-Kansas City, Kansas City, Missouri, USA

**Keywords:** extracellular matrix, proteoglycan, tenascin, receptor protein tyrosine phosphatase, perineuronal net, plasticity, protein–protein interaction, X-ray crystallography

## Abstract

Neural plasticity, the ability to alter the structure and function of neural circuits, varies throughout the age of an individual. The end of the hyperplastic period in the central nervous system coincides with the appearance of honeycomb-like structures called perineuronal nets (PNNs) that surround a subset of neurons. PNNs are a condensed form of neural extracellular matrix that include the glycosaminoglycan hyaluronan and extracellular matrix proteins such as aggrecan and tenascin-R (TNR). PNNs are key regulators of developmental neural plasticity and cognitive functions, yet our current understanding of the molecular interactions that help assemble them remains limited. Disruption of *Ptprz1*, the gene encoding the receptor protein tyrosine phosphatase RPTPζ, altered the appearance of nets from a reticulated structure to puncta on the surface of cortical neuron bodies in adult mice. The structural alterations mirror those found in *Tnr*^*−/−*^ mice, and TNR is absent from the net structures that form in dissociated cultures of *Ptprz1*^*−/−*^ cortical neurons. These findings raised the possibility that TNR and RPTPζ cooperate to promote the assembly of PNNs. Here, we show that TNR associates with the RPTPζ ectodomain and provide a structural basis for these interactions. Furthermore, we show that RPTPζ forms an identical complex with tenascin-C, a homolog of TNR that also regulates neural plasticity. Finally, we demonstrate that mutating residues at the RPTPζ–TNR interface impairs the formation of PNNs in dissociated neuronal cultures. Overall, this work sets the stage for analyzing the roles of protein–protein interactions that underpin the formation of nets.

The extracellular matrix (ECM) of the central nervous system (CNS) is structurally and functionally unique. In contrast to other organ systems, the neural ECM contains relatively small amounts of typical fibrous ECM proteins. Instead, it is highly enriched in glycosaminoglycans (GAGs) and a unique assortment of proteoglycans and glycoproteins (1). Over the last 2 decades, there has been rapidly growing interest for the critical role the neural matrix plays in neural development and functions. And yet, because of its unique composition, our current understanding of the role that the neural matrix plays in the central nervous system exceeds our grasp of its molecular architecture. This disconnect is perhaps best exemplified by the CNS-specific ECM structures called perineuronal nets (PNNs).

PNNs are lattice-like structures surrounding subsets of neurons exclusively in the CNS that were first described over a century ago (2). The formation of PNNs is particularly intriguing because in areas such as the cerebral cortex, they form coincident with the maturation of the brain from a hyperplastic juvenile state to a more stable and less plastic mature state. For example in the visual cortex, PNNs are most prominently expressed surrounding parvalbumin-expressing interneurons, which are critically involved in closing the period of hyperplasticity early in neural development called the critical period (3). Furthermore, PNNs are expressed in an activity-dependent manner coincident with the closure of the critical period for ocular dominance plasticity—the dominance of the visual functions of one eye over the other (4). These and other findings led to the hypothesis that PNNs are key regulators of developmental plasticity in the CNS (4). In line with these observations, it was demonstrated that disruption of PNNs is sufficient to reopen the critical period for ocular dominance plasticity in the visual cortex (5). The roles of PNNs extend beyond controlling plasticity in sensory systems, and these structures have now been linked to the acquisition of memories (6, 7), including fear learning in the amygdala. Therefore, PNNs are established critical regulators of developmental plasticity in the CNS.

Although the specific composition of PNNs vary throughout the brain (8), nets are generally composed of the following: (i) the GAG hyaluronan, (ii) the ECM protein aggrecan (ACAN) as well as other members of the lectican family of chondroitin sulfate proteoglycans (9), (iii) the secreted glycoprotein proteoglycan link protein (HAPLN1) (10), and (iv) tenascin-R (TNR) (11). These carbohydrate and protein components assemble to form the honeycomb structure that is characteristic of PNNs (2). Genetic ablation of the PNN components ACAN (12), HAPLN1 (10), or TNR (11) disrupts the formation of PNNs and alters plasticity, although precise alteration of PNN structures through manipulation of these components remains limited. Furthermore, one of the most puzzling aspects of PNN assembly is that the components found in nets are also found in other substructures within the neural matrices. For this reason, the extent to which all the critical components of nets have been identified and how their interactions initiate the formation of nets around specific neuronal populations remain undetermined.

Recent work extended our understanding of PNN structure by demonstrating that the chondroitin sulfate proteoglycan phosphacan is also a critical component of PNNs (13, 14). Phosphacan is a secreted variant of protein tyrosine phosphatase receptor type Z (PTPRZ/RPTPζ encoded by *PTPRZ1*) generated by alternative splicing or, alternatively, by cleavage of the full-length receptor form of the protein. It includes the extracellular region of the receptor along with a ∼860 amino acid insert, featuring attachment sites for chondroitin sulfate GAGs (13). Genetic disruption of the gene encoding RPTPζ altered the appearance of nets from a reticulated structure to puncta on the surface of cortical neuron bodies in adult animals (15). Interestingly, the discontinuous aspect of nets in adult *Ptprz1*^−/−^ mice matches the disruptions observed in *Tnr*^−/−^ mice. Furthermore, the net structures that form in dissociated cultures of *Ptprz1*^−/−^ cortical neurons have dramatically reduced levels of TNR (15). Taken together, these findings suggest that TNR and RPTPζ might function in the same pathway to promote the assembly of PNNs.

Physical interactions between TNR and RPTPζ/phosphacan have only been characterized to a limited extent (16). In contrast, several groups have reported that RPTPζ associates with a homolog of TNR called tenascin-C (TNC) that is also found in the CNS (17, 18). TNC appears to be a minor component of PNNs (19). Unlike *Tnr*, removal of *Tnc* does not disrupt the assembly of nets though it still leads to a decrease in plasticity (20). In addition, severe defects in the formation of PNNs are observed in the cultures of hippocampal neurons from mice lacking TNC, TNR, and the lecticans brevican and neurocan (21). The fact that (i) we observed similar alterations in the appearance of PNNs in *Tnr*^−/−^ and *Ptprz1*^−/−^ adult mice, (ii) the proteins TNR and TNC share a similar architecture (22), and (iii) RPTPζ has been reported to interact physically with TNR and TNC led us to the hypothesis that the formation of a complex between RPTPζ and TNR plays a critical role in the assembly of nets and that the interactions between RPTPζ and TNR/TNC occur through similar interfaces.

Here, we demonstrate that TNR and TNC associate with the single fibronectin type III (FN) domain found in RPTPζ and provide a structural basis of these interactions. Furthermore, our experiments suggest that mutating TNR-binding residues in RPTPζ impairs the formation of PNNs. Overall, our findings provide novel structural insights in the interactions between RPTPζ and tenascin family members, prove that the formation of a RPTPζ–TNR complex is essential to the assembly of PNNs, and provide a blueprint to explore a potential role for a RPTPζ–TNC complex in neural function.

## Results

### The single FN repeat found in RPTPζ interacts with TNC and TNR

**Figure 1:**
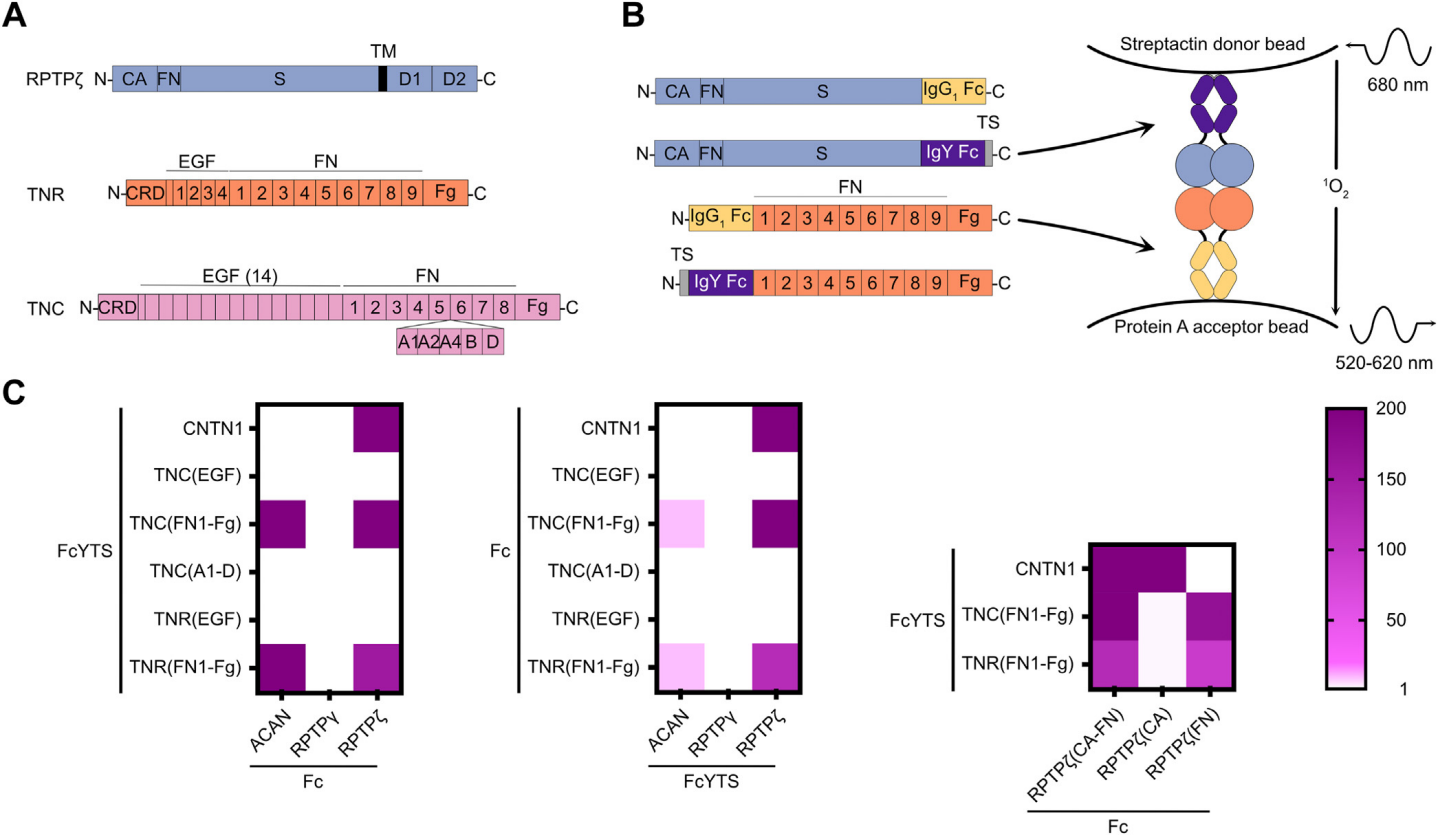
**Interactions between RPTPζ and the homologous TNC and TNR.***A*, overview of the domain organization of RPTPζ, TNR, and TNC. RPTPζ includes a carbonic anhydrase-like (CA) domain, a single fibronectin type III domain (FN), a spacer region, and two cytoplasmic tyrosine phosphatase domains. The splice form of RPTPζ called phosphacan does not include the transmembrane or cytoplasmic region but include a large segment featuring multiple glycosaminoglycan attachment sites in addition to the spacer region. TNR includes a cysteine-rich domain (CRD), four EGF repeats, nine FN domains, and a C-terminal fibrinogen-like (Fg) domain. A segment akin to “half” of an EGF repeat is inserted between the CRD and the first full EGF repeat. TNC includes a CRD and 14 EGF repeats separated by a segment that is akin to “half” of an EGF repeat. TNC also features eight FN domains and a C-terminal Fg domain. Multiple splice forms of TNC exist that include manifold combinations of FN repeats between FN domains 5 and 6. Here, repeats A1, A2, A4, B, and D are shown. *B*, design of constructs used in the binding assay using RPTPζ and TNR as examples. The ectodomain of RPTPζ and regions of TNR (here, the FN1-Fg domains) are fused to the Fc domain of human IgG1 or a protein designated FcYTS that includes the following: (1) the Cν3 and Cν4 domains of chicken IgY and (2) a Twin-Strep tag (TS) for detection with streptactin. Proteins are expressed in HEK293 cells and conditioned media is used for experiments. Potential interactions between the Fc and FcYTS candidate proteins are tested in an Alpha-binding assay by mixing conditioned media including Fc and FcYTS fusion proteins along with streptactin donor beads and protein A-acceptor beads. In AlphaScreen assays, excitation of donor beads at 680 nm triggers the release of a singlet oxygen. An acceptor bead hit by this highly reactive molecule emits a signal between 520 and 620 nm. Because the half-life of the singlet oxygen is limited, a luminescent signal is only obtained when the donor beads and acceptor beads are within 200 nm. Here, the binding of the FcYTS fusion protein of RPTPζ immobilized on donor beads to the Fc fusion protein of TNR(FN1-Fg) bound to acceptor beads brings the two beads in proximity. *C*, results of the binding assay shown in a heat map representation. The scale indicates the value of the signal for the protein pair protein 1-FcYTS/protein 2-Fc divided by the signal measured for the protein 1-FcYTS/Fc only pair. Accordingly, these calculated values are unitless. Raw signals are included in Table S1. CA, carbonic anhydrase-like; CRD, cysteine-rich domain; EGF, epidermal growth factor-like; Fg, fibrinogen-like; FN, fibronectin type III; FN1, first FN repeat; RPTPγ, protein tyrosine phosphatase receptor type G; RPTPζ, protein tyrosine phosphatase receptor type Z; TNC, tenascin-C; TNR, tenascin-R.

TNC and TNR share a common domain organization that includes an N-terminal cysteine-rich region responsible for the formation of disulfide-linked multimers, followed by 5-14 epidermal growth factor (EGF)-like domains, 8 to 9 FN repeats, and a C-terminal fibrinogen (Fg)-like domain (Fig. 1*A*). The number of EGF and FN repeats differ between TNC and TNR, yet the main difference between these two homologous proteins is the inclusion of multiple combinations of FN domains between repeats FN5 and FN6 of TNC through alternative splicing (23). Although multiple lines of evidence indicate that TNR and its homolog TNC interact with RPTPζ, the locations of the RPTPζ-binding sites remain unclear (16, 17, 18). Indeed, the EGF-like repeats of TNR have been implicated in associating with phosphacan (16), while both the C-terminal Fg domain of TNC and the alternatively spliced FN repeats A1, A2, and A4 have been reported to bind to RPTPζ (17, 18). Given the sequence homology between TNR and TNC (22), we reasoned that RPTPζ would bind to a region conserved between these two proteins and decided to reevaluate the interactions between RPTPζ, TNR, and TNC. A binding assay previously designed to characterize the interactions between amyloid precursor protein and contactin family members was thus adapted to accomplish this goal (24).

These binding assays were developed using AlphaScreen technology in which a luminescent signal is emitted when a protein bound to a donor bead associates to another protein bound to an acceptor bead. In our case, regions of TNC, TNR, or the human RPTPζ ectodomain were expressed transiently in HEK293 as fusion proteins with the Fc region of human IgG1 or the Fc region of chicken IgY along with a Twin-Strep tag (25) (Fig. 1*B*). While proteins fused to human IgG1 bind to protein A acceptor beads, proteins fused to the IgY-Twin-Strep tag domain (designated FcYTS) bind to donor beads coated with streptactin, a mutant of streptavidin engineered to associate with the Twin-strep tag. Binding of proteins with these two distinct tags brings the beads in close proximity, which is followed by emission of a luminescent signal (Fig. 1*B*).

The assay design was first validated by testing previously characterized interactions between RPTPζ and contactin-1 (CNTN1) (26, 27) and between TNC, TNR, and ACAN (28) as positive controls in two distinct “orientations”: Fc *versus* FcYTS and FcYTS *versus* Fc. We successfully detected binding between the pairs RPTPζ-Fc/CNTN1-FcYTS and RPTPζ-FcYTS/CNTN1-Fc; Fc-TNC(FN1Fg)/FcYTS-ACAN and FcYTS-TNC(FN1Fg)/Fc-ACAN; as well as Fc-TNR(FN1Fg)/FcYTS-ACAN and FcYTS-TNR(FN1Fg)/Fc-ACAN (Fig. 1*C*). Our methodology validated, we then evaluated the ability of RPTPζ to bind to regions of TNC and TNR. We could not detect interactions between RPTPζ and the EGF repeats of TNR or the alternatively spliced repeats A1, A2, A4, B, and D of TNC. However, domains FN1-Fg of TNC and TNR bound to RPTPζ while they did not associate with RPTPγ, a homolog of RPTPζ (29). Subsequent deletion experiments indicated that TNC and TNR bound specifically to the single FN repeat found in the extracellular region of RPTPζ (Fig. 1*C*).

### The C-terminal Fg domains of TNC and TNR associate with RPTPζ with similar affinities

**Figure 2:**
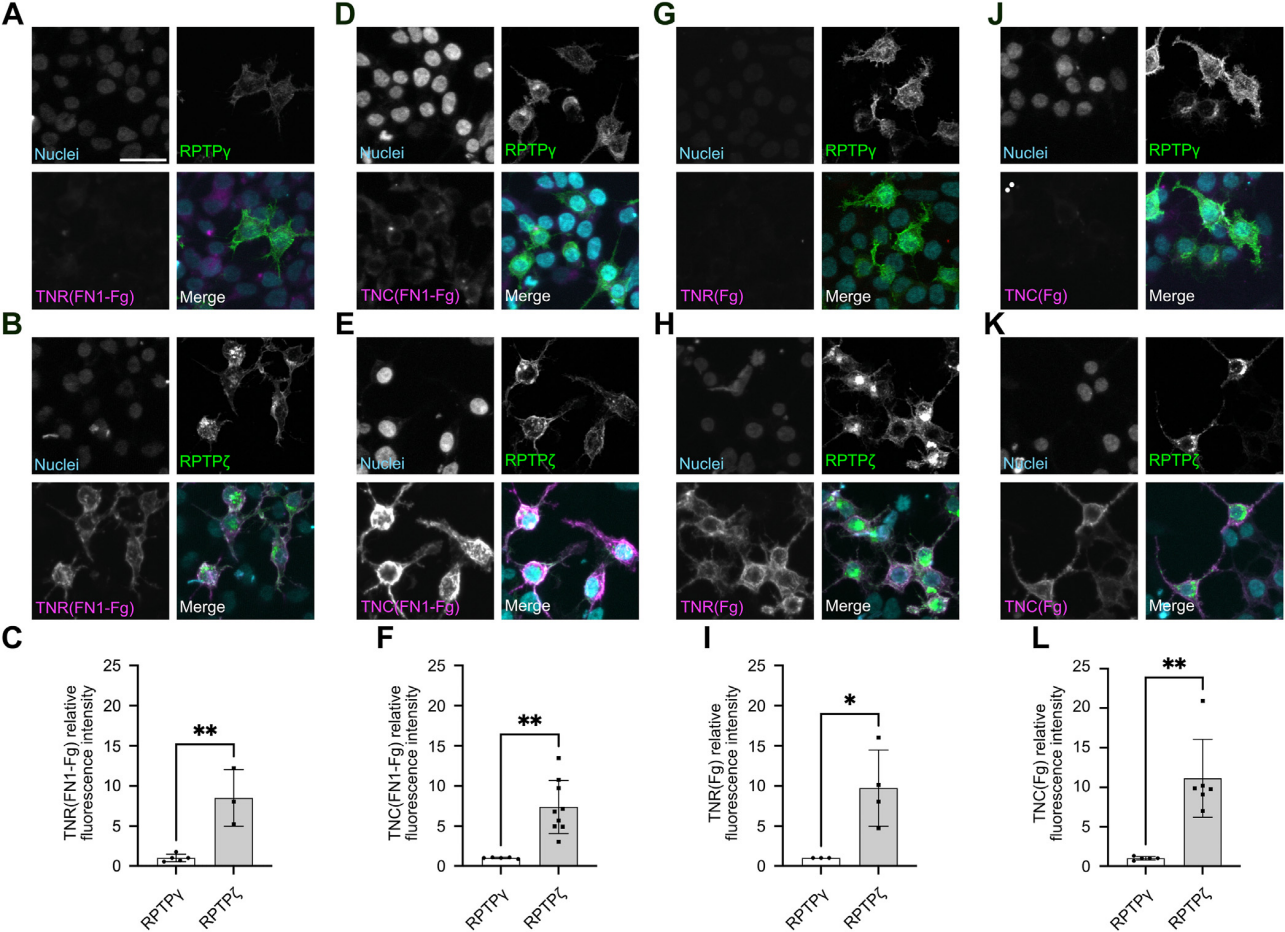
**The C-terminal Fg domains of TNR and TNC associate with RPTPζ-transfected cells.***A* and *B*, a fusion protein of human IgG1 Fc and the FN1-Fg domains of human TNR binds to HEK293 cells transfected with an Emerald-RPTPζ fusion protein, but not to cells transfected with Emerald-RPTPγ. Transfected cells were incubated with a complex of domains FN1-Fg of human TNR fused to human IgG Fc with goat anti-human IgG conjugated to Alexa Fluor 568. Transfected RPTPγ and RPTPζ were detected using the Emerald fusion partner. Nuclei were visualized with DAPI. The experiment was performed three times, one representative set of images is shown. Scale bar represents 25 μm. *C*, fold differences of the fluorescent intensity of cell surface staining for TNR(FN1-Fg) between Emerald-RPTPγ-expressing (*A*) and Emerald-RPTPζ-expressing (*B*) cells normalized to Emerald-RPTPγ–expressing cells. ∗∗*p* = 0.0025. *D* and *E*, a fusion protein of human IgG1 Fc and the FN1-Fg domains of human TNC binds to HEK293 cells transfected with Emerald-RPTPζ, but not to cells transfected with Emerald-RPTPγ. Transfected cells were incubated with a complex of domains FN1-Fg of human TNC fused to human IgG Fc with goat anti-human IgG conjugated to Alexa Fluor 568. Transfected RPTPγ and RPTPζ were detected using the Emerald fusion partner. Nuclei were visualized with DAPI. The experiment was performed three times, one representative set of images is shown. *F*, fold differences of the fluorescent intensity of cell surface staining for TNC(FN1-Fg) between Emerald-RPTPγ–expressing (*D*) and Emerald-RPTPζ–expressing (*E*) cells normalized to Emerald-RPTPγ–expressing cells. ∗∗*p* = 0.0012. *G* and *H*, a fusion protein of human IgG1 Fc and the Fg domain of human TNR binds to HEK293 cells transfected with Emerald-RPTPζ, but not to cells transfected with Emerald-RPTPγ. Transfected cells were incubated with a complex of the Fg domain of human TNR fused to human IgG Fc with goat anti-human IgG conjugated to Alexa Fluor 568. Transfected RPTPγ and RPTPζ were detected using the Emerald fusion partner. Nuclei were visualized with DAPI. The experiment was performed three times, one representative set of images is shown. *I*, fold differences of the fluorescent intensity of cell surface staining for TNR(Fg) between Emerald-RPTPγ–expressing (*G*) and Emerald-RPTPζ–expressing (*H*) cells normalized to Emerald-RPTPγ–expressing cells. ∗*p* = 0.0266. *J* and *K*, a fusion protein of human IgG1 Fc and the Fg domain of human TNC binds to HEK293 cells transfected with Emerald-RPTPζ, but not to cells transfected with Emerald-RPTPγ. Transfected cells were incubated with a complex of the Fg domain of human TNC fused to human IgG Fc with goat anti-human IgG conjugated to Alexa Fluor 568. Transfected RPTPγ and RPTPζ were detected using the Emerald fusion partner. Nuclei were visualized with DAPI. The experiment was performed three times, one representative set of images is shown. *L*, fold differences of the fluorescent intensity of cell surface staining for TNC(Fg) between Emerald-RPTPγ–expressing (*J*) and Emerald-RPTPζ–expressing (*K*) cells normalized to Emerald-RPTPγ–expressing cells. ∗∗*p* = 0.0014. Note that in all conditions there were no statistical differences in the fluorescent intensity of Emerald-RPTPγ or Emerald-RPTPζ between cells. Individual data points are represented by *black circles* and *squares* on all graphs. Error bars represent the SD of the mean. DAPI, 4′,6-diamidino-2-phenylindole; Fg, fibrinogen-like; FN1, first FN repeat; RPTPγ, protein tyrosine phosphatase receptor type G; RPTPζ, protein tyrosine phosphatase receptor type Z; TNC, tenascin-C; TNR, tenascin-R.

Cell surface binding assays were undertaken next to determine whether interactions between RPTPζ and tenascins could occur on the surface of cells. We first assessed the extent to which domains FN1-Fg of TNR fused to human IgG could associate with full-length RPTPζ expressed in HEK293 cells with an Emerald fluorescent tag at its N-terminus (Fig. 2, *A*–*C*). In these experiments, TNR(FN1-Fg) bound to cells transfected with Emerald-RPTPζ (Fig. 2*B*), but not the homologous Emerald-RPTPγ (Fig. 2*A*). Similar results were obtained with domains FN1-Fg of human TNC (Fig. 2, *D*–*F*). Because the C-terminal Fg domain had previously been implicated in binding RPTPζ (17), we asked whether the Fg domains of TNC and TNR could mediate interactions with RPTPζ. As was the case for FN1-Fg, the Fg domains of TNR (Fig. 2, *G*–*I*) and TNC (Fig. 2, *J*–*L*) fused to human IgG1 Fc bound specifically to Emerald-RPTPζ. Taken together, these findings suggest that TNC and TNR interact specifically with RPTPζ at the surface of cells and that this interaction is mediated by their respective C-terminal Fg domains.

**Figure 3:**
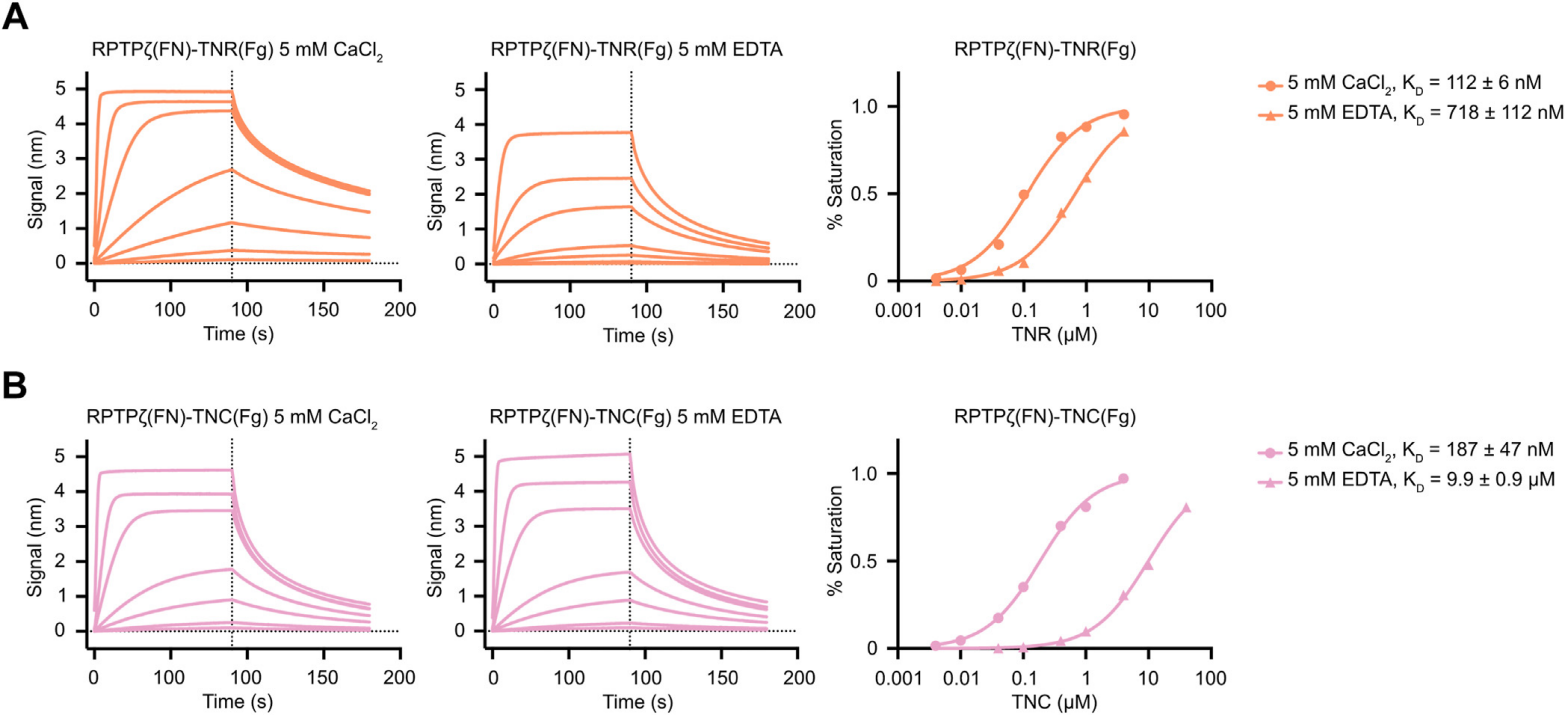
**Characterization of RPTPζ binding to TNR and TNC by biolayer interferometry.***A*, the FN domain of RPTPζ was biotinylated in a 1:1 molar ratio, immobilized onto streptavidin sensors, and titrated against 4 μM, 1 μM, 400 nM, 100 nM, 40 nM, 10 nM, 4 nM of the Fg domain of human TNR in the presence of CaCl_2_ or EDTA. The *left* and *center* panels show representative association and dissociation curves with the *vertical dashed lines* indicating the start of the dissociation phase. The *right panel* shows the concentration-response plots obtained from these sensorgrams and normalized to a maximum response. K_D_ values are reported as averages ± SDs from at least four experiments. Additional information about individual experiments used in affinity calculations are listed in Table S1. *B*, the FN domain of RPTPζ was biotinylated in a 1:1 molar ratio, immobilized onto streptavidin sensors, and titrated against varying concentrations of the Fg domain of human TNC. The concentration range was 4 μM, 1 μM, 400 nM, 100 nM, 40 nM, 10 nM, 4 nM for experiments carried out in the presence of CaCl_2_, while the range was 40μM, 10 μM, 4 μM, 1 μM, 400 nM, 100 nM, 40 nM for experiments conducted in the presence of EDTA. The *left* and *center* panels show representative association and dissociation curves with the *vertical dashed lines* indicating the start of the dissociation phase. The *right panel* shows the concentration-response plots obtained from these sensorgrams and normalized to a maximum response. K_D_ values are reported as averages ± SDs from at least four experiments. Additional information about individual experiments used in affinity calculations are listed in Table S1. Fg, fibrinogen-like; FN, fibronectin type III; RPTPζ, protein tyrosine phosphatase receptor type Z; TNC, tenascin-C; TNR, tenascin-R.

Finally, the interactions between RPTPζ and tenascins were characterized using bio-layer interferometry (BLI) by measuring the binding between the biotinylated FN domain of RPTPζ immobilized on streptavidin sensors and the Fg domain of TNR or TNC (Fig. 3). The binding affinity (K_D_) was calculated by plotting the maximal signal measured at equilibrium for a series of TNR concentrations. Because Fg domains include a bound Ca^2+^ ion (30), we conducted assays in the presence of 5 mM CaCl_2_ or 5 mM EDTA. We measured a K_D_ of 112 nM in the presence of Ca^2+^ ions, while the removal of divalent cations reduced the affinity of the interaction six-fold (K_D_ = 712 nM). The measured affinities and the apparent lack of Ca^2+^ dependance are in agreement with early work identifying interactions between TNR and phosphacan (31). In the case of TNC, the dissociation constant was similar to the one measured for RPTPζ–TNR interactions (K_D_ = 187 nM). Unlike what was observed with TNR, the interactions with TNC were significantly weakened upon removal of Ca^2+^ ions (K_D_ = 9.9 μM, fifty-fold reduction), in line with a previously published report (17).

### Three residues in the FN domain of RPTPζ are essential to interact with TNR

**Figure 4:**
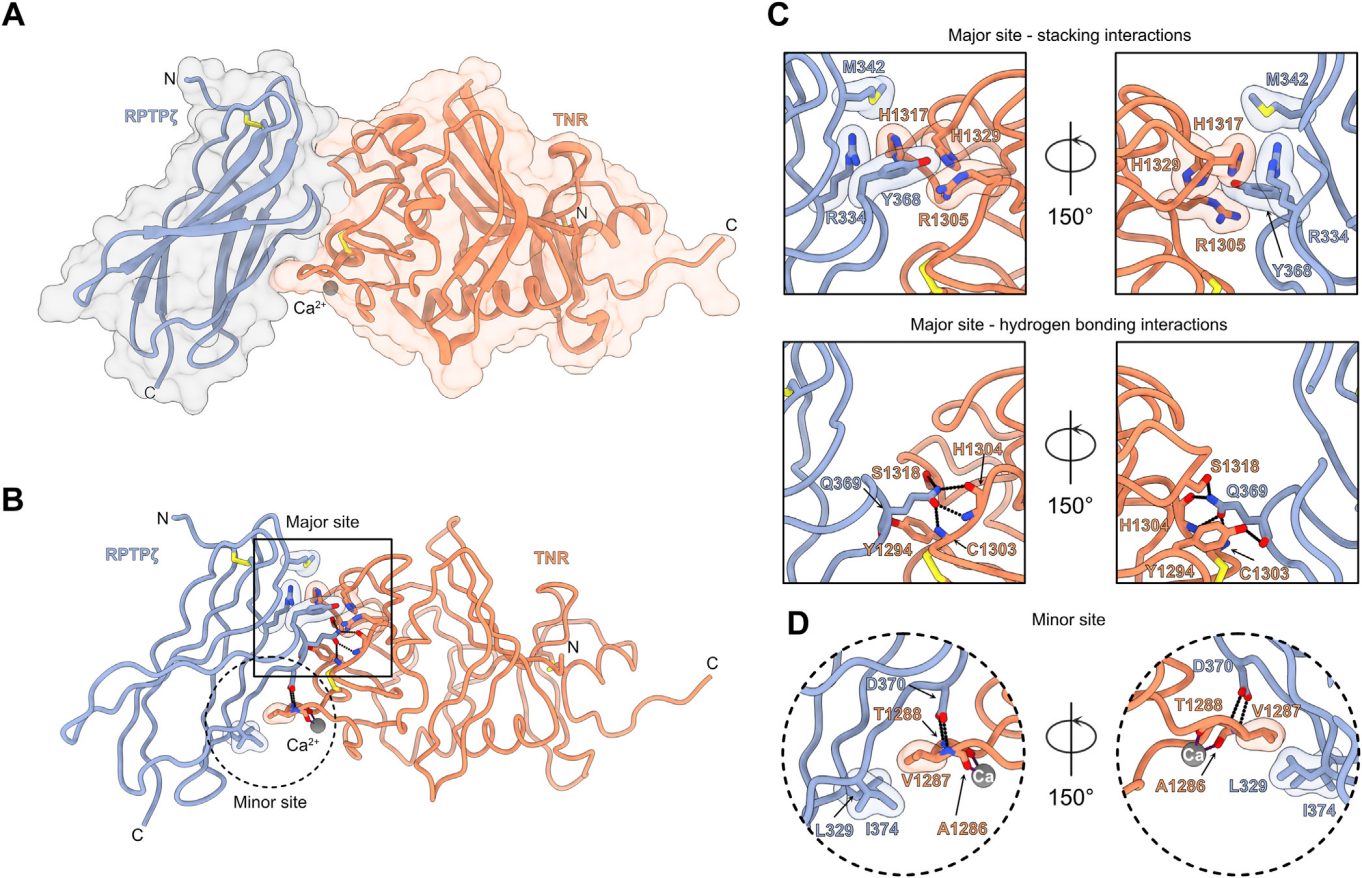
**Crystal structure of the RPTPζ-TNR complex.***A*, the RPTPζ–TNR complex is shown in ribbon diagram overlaid on translucent surfaces. The FN domain of RPTPζ is colored *slate*, while the Fg domain of TNR is shown in *salmon*. The position of a Ca^2+^ ion bound to TNR is indicated by a *gray sphere*. The letters N and C indicate the N- and C-termini, respectively. *B*, the complex interface can be divided into a major binding site (*solid line square*) and a minor binding site (*dotted line circle*). Contacting residues are represented as *sticks*, while transparent surfaces denote residues involved in van der Waals or packing interactions. Potential hydrogen bonds are represented as *dashed lines* between interacting atoms. *C*, detailed view of the interactions in the major site. For clarity, only stacking interactions are shown on the two *top panels* while the hydrogen bonding residues are shown only in the two *bottom panels*. In these panels, interacting atoms of side chains or main chain atoms are displayed only if they participate in the interactions. *D*, detailed view of the interactions at the minor site. The *gray* sphere denotes the Ca^2+^ ion bound to the Fg domain of TNR. Fg, fibrinogen-like; FN, fibronectin type III; FN1, first FN repeat; RPTPζ, protein tyrosine phosphatase receptor type Z; TNR, tenascin-R.

We sought to obtain atomic-level insights between RPTPζ and TNR to gain further information into the function of the complex. Purified RPTPζ(FN) and TNR(Fg) were thus mixed in a 1:1: molar ratio prior to carrying out crystallization trials. The complex structure was solved by molecular replacement using the crystal structures of the Fg domain of tachylectin 5A and the FN5 domain of TNC as search models (32, 33), and the final model was refined to 1.8 Å (R_work_/R_free_ = 0.174/0.210, Table 1, Figs. 4*A* and S1). There are four complexes in the asymmetric unit that adopt a similar arrangement and can be superimposed with RMSD values ranging from 0.413 Å to 0.728 Å. Averaging over of the four complex molecules in the unit cell, the RPTPζ/TNR interface buries 496 Å^2^ on TNR and 535 Å^2^ RPTPζ for a modest total surface area of 1031 Å^2^. However, the shape complementarity coefficient of 0.74 suggests a highly complementary interface between the two proteins (34). The Fg region of TNR includes a single Ca^2+^ ion (Fig. 4*A*), and comparison of the bound and free form of human TNR(Fg) indicates that formation of the complex does not induce any significant structural rearrangement (Fig. S2). The FN region of RPTPζ adopts the canonical β-sandwich fold described for other FN domains (33), although it features an unusual disulfide bridge between C311 and C392. A loop protruding from the FN domain of RPTPζ contacts a shallow groove at the surface of TNR(Fg).

**Table 1:** Data collection and refinement statistics

Crystal	Human RPTPζ-TNR	Human TNR(Fg)	Human RPTPζ-TNC	Human TNC(Fg) (Y2140H, S2164H)
Data collection				
Beamline	APS 22-ID	APS 22-BM	APS 22-ID	APS 22-ID
Wavelength (Å)	1.00	1.00	1.00	1.00
Number of unique reflections	102,222 (10,193)	27,862 (2576)	25,470 (2506)	46,455 (4286)
Resolution (Å)	50.00–1.80	50.00–1.75	50.00–1.89	50.00–1.80
Space group	P1	P3_1_21	P2_1_2_1_2_1_	C2
Unit cell				
a, b, c (Å)	48.33, 75.66, 88.32	47.42, 47.42, 203.40	54.71, 61.52, 92.43	106.05, 101.56, 56.67
α, β, γ (º)	104.41, 92.40, 91.81	90.00, 90.00, 120.00	90.00, 90.00, 90.00	90.00, 122.35, 90.00
R_merge_	0.157 (0.991)	0.128 (0.491)	0.113 (0.342)	0.068 (0.250)
R_pim_	0.062 (0.517)	0.06 (0.240)	0.054 (0.172)	0.028 (0.104)
Completeness (%)	91.6 (91.1)	99.2 (94.9)	99.6 (99.2)	98.9 (91.5)
Redundancy	6.6 (3.9)	5.7 (4.6)	4.9 (4.3)	6.9 (6.3)
I/σI	11.7 (1.1)	10.1 (2.7)	16.2 (4.7)	20.8 (4.8)
C/C_1/2_	0.988 (0.486)	0.985 (0.845)	0.994 (0.949)	0.992 (0.987)
Refinement				
PDB code	8FN9	8FNA	8FN8	8FNB
Number of protein chains in asymmetric unit	8	1	2	2
Resolution (Å)	42.96–1.80	38.08–1.75	40.88–1.89	34.83–1.80
Reflections (Test)	102,103 (2178)	27,793 (2001)	25,084 (1979)	44,562 (1915)
R_work_^c^/R_free_	0.174/0.210	0.160/0.185	0.164/0.205	0.152/0.174
Number of nonhydrogen atoms	11,035	2062	2836	3999
Protein	10,459	1815	2606	3554
Ligand	4	7	1	12
Water	572	240	229	433
R.m.s.d				
Ideal bonds (Å)	0.011	0.013	0.003	0.009
Ideal angles (°)	0.877	1.131	0.667	0.872
Average B factors (Å^2^)	35.4	29.3	26.2	29.7
Protein	35.3	28.2	25.7	28.8
Ligand	31.7	26.3	32.0	47.5
Water	37.7	37.8	19.7	36.9
Ramachandran statistics				
Favored (%)	95.84	96.24	95.22	95.16
Allowed (%)	4.16	3.76	4.78	4.84
Rotamer outlier (%)	0.18	0.00	0.36	0.55

Values in parentheses apply to the high-resolution shell.

Broadly, the RPTPζ–TNR interface can be divided into a major contact site and a minor contact site, and the following description only details contacts common to all complexes in the unit cell (Fig. 4*B*). At the major contact site, R334, M342 in RPTPζ pack against H1317 in TNR, while Y368 stacks against R1305, H1317, and H1329 (Fig. 4*C*). The major contact site also includes a network of hydrogen bonding interactions that are mediated exclusively by Q369 in RPTPζ. Specifically, the side chain amide group of Q369 forms a hydrogen bond with the hydroxyl group of S1318 and with the main chain nitrogen and oxygen atoms of H1304 and the amino group of C1303. Furthermore, the hydroxyl group of Y1294 forms a hydrogen bond with the carbonyl group of Q369. Finally, in the minor interaction site (Fig. 4*D*), the side chains of RPTPζ residues L329 and I374 are wedged against the aliphatic side chain of V1287 in TNR, while the side chain carboxylate atoms D370 form hydrogen bonds with the main chain nitrogen atoms of V1287 and T1288. The Ca^2+^ ion is located close to the minor RPTPζ-binding site, although it does not participate directly in interactions with the FN domain. In particular, the carbonyl atoms of A1286 and T1288 coordinate the Ca^2+^ ion. As such, removal of Ca^2+^ may disrupt contacts in the minor site but not interfere significantly with residues in the major binding site. This hypothesis is consistent with the modest decrease in binding affinity that was measured after removal of divalent cations (Fig. 3*A*).

Interestingly, the Fg domain of TNR matches closely with the one found in the lectin tachylectin 5A (RMSD of 1.7 Å over 210 Cα positions) in spite of only 46% sequence identity at the amino acid level (Fig. S3). Furthermore, the major contact site in TNR(Fg) overlaps with the N-acetyl-D-glucosamine–binding site found in the Fg domain of tachylectin 5A (Fig. S3), and the amide group of Q369 in RPTPζ(FN) occupies a position identical to the acetyl group in the bound carbohydrate (Fig. S3*C*). Consistent with this observation, residues in the major contact site of TNR(Fg) are equivalent to those that interact with the N-acetyl-D-glucosamine in the lectin. These findings indicate that the Fg domain can accommodate structurally unrelated interactors within the same binding region. In fact, this site is also used by the C-terminal Fg domain in fibrinogen to interact with the α chain of fibrin or by the Fg module of angiopoietin-2 to interact with the receptor tyrosine kinase Tie2 (35, 36).

**Figure 5:**
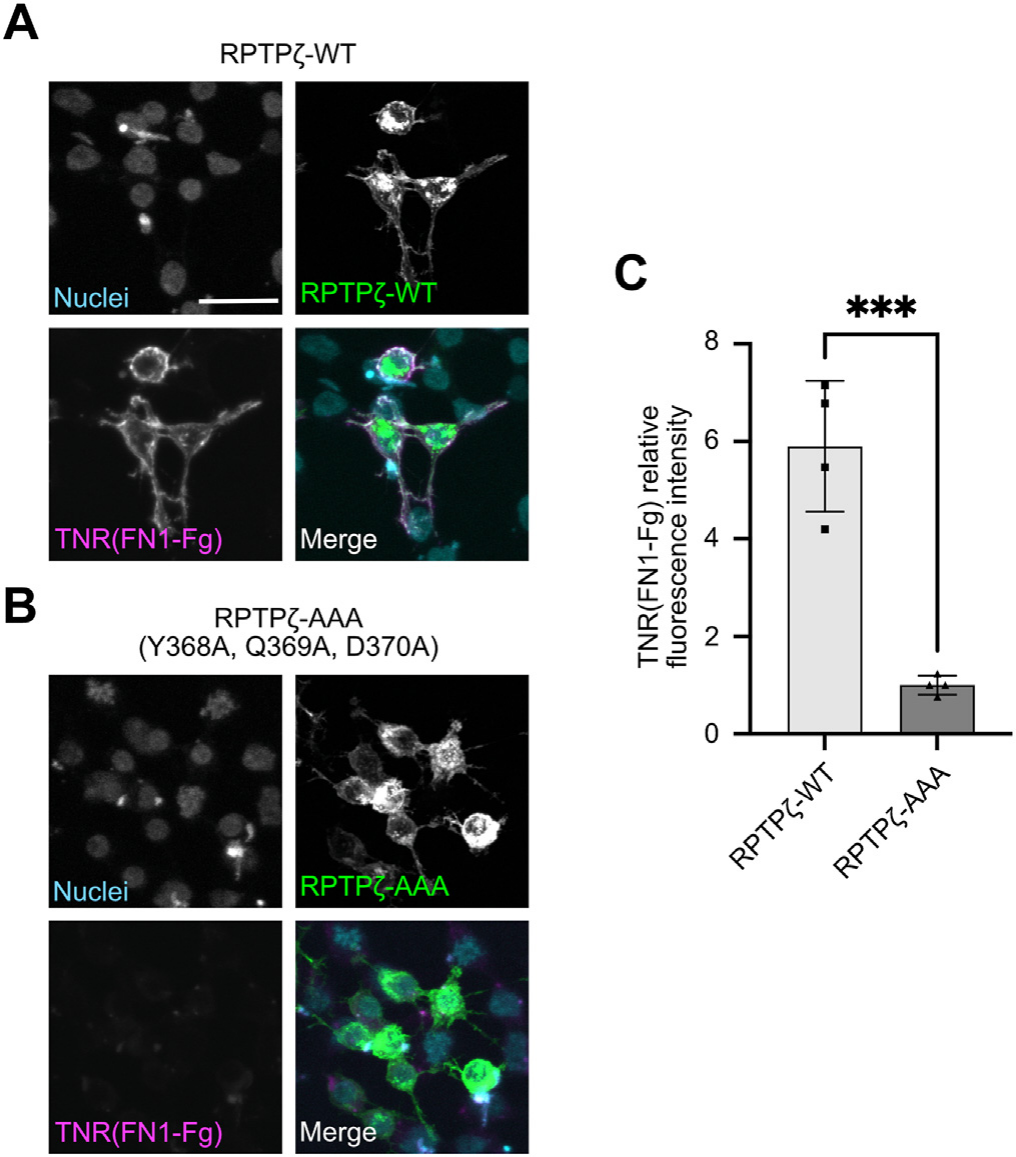
**Alanine substitutions at the RPTPζ–TNR interface prevent interactions between RPTPζ and TNR.***A*, a fusion protein of human IgG1 Fc and the FN1-Fg domains of human TNR binds to HEK293 cells transfected with an Emerald-RPTPζ fusion protein. RPTPζ was detected on the surface of cells using its N-terminal Emerald fluorescent tag, while TNR is bound to a goat anti-human IgG antibody conjugated to Alexa Fluor 568. The nuclei were visualized using DAPI. The experiment was performed three times, one representative set of images is shown. Scale bar represents 25 μM. *B*, a fusion protein of human IgG1 Fc and the FN1-Fg domains of human TNR does not bind to HEK293 cells transfected with an Emerald-RPTPζ fusion protein in which residues Y368, Q369, and D370 are changed to alanine. RPTPζ and bound TNR were detected as described in *panel A*. The nuclei were visualized using DAPI. The experiment was performed three times, one representative set of images is shown. *C*, fold differences of the fluorescent intensity of cell surface staining for TNR(FN1-Fg) between Emerald-RPTPζ-WT–expressing (*A*) and Emerald-RPTPζ-WT-AAA–expressing (*B*) cells normalized to the Emerald-RPTPζ-WT-AAA–expressing cells. ∗∗∗*p* = 0.0004. There was no statistical difference in the fluorescent intensity of Emerald-RPTPζ-WT and Emerald-RPTPζ-WT-AAA between cells. Individual data points are represented by *black squares* and *triangles*. Error bars represent the SD of the mean. DAPI, 4′,6-diamidino-2-phenylindole; Fg, fibrinogen-like; FN, fibronectin type III; FN1, first FN repeat; RPTPζ, protein tyrosine phosphatase receptor type Z; TNR, tenascin-R.

Finally, cell surface binding assays were used to validate that the complex interface identified in the RPTPζ–TNR complex structure is relevant to interactions *in situ* (Fig. 5). Residues Y368, Q369, and D370 mediate the majority of the contacts between RPTPζ and TNR, so they were mutated to alanine in Emerald-RPTPζ to test whether this protein retained the ability to bind to TNR(FN1-Fg). HEK293 cells were transfected with the WT and mutated form of Emerald-RPTPζ and incubated with a Fc fusion protein of TNR(FN1-Fg). As was observed previously (Fig. 2, *A*–*C*), TNR bound specifically to Emerald-RPTPζ, but introduction of the three alanine mutations prevented these interactions (Fig. 5). The sum of these analyses shows that RPTPζ binds to TNR through a small binding interface centered around Y368, Q369, and D370. Because Y368 and Q369 are replaced by D and K, respectively, in RPTPγ, these results also explain the lack of interaction between RPTPγ and TNR.

### Interactions between RPTPζ and TNC mirror the ones found in the RPTPζ–TNR complex

**Figure 6:**
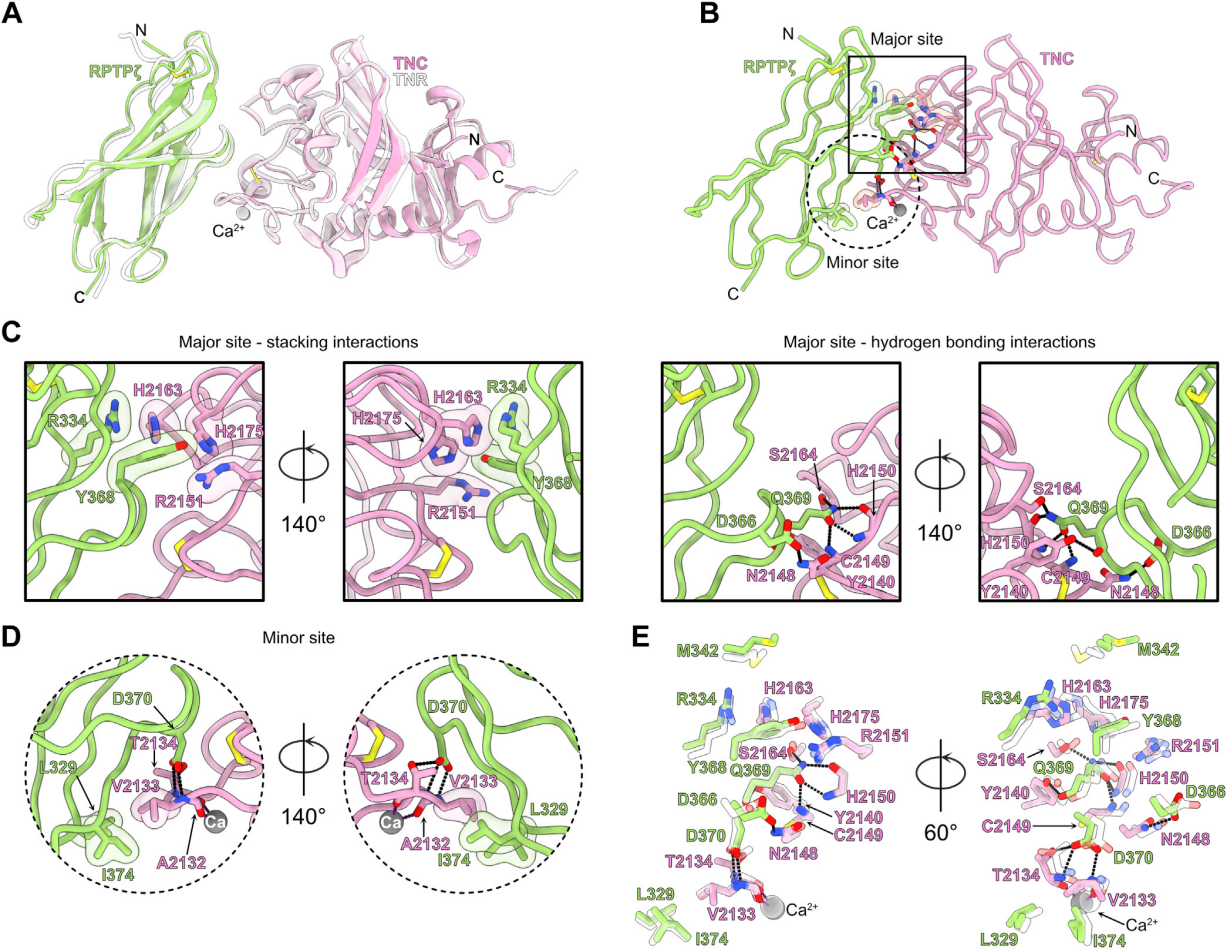
**Crystal structure of the RPTPζ–TNC complex and comparison with the RPTPζ–TNR complex.***A*, the RPTPζ–TNC complex is shown in ribbon diagram overlaid onto the RPTPζ-TNR (colored *white*). The FN domain of RPTPζ is colored *green*, while the Fg domain of TNC is shown in *pink*. The position of Ca^2+^ ions bound to TNC and TNR is indicated by *gray* and *white transparent spheres*, respectively. The letters N and C indicate the N- and C-termini, respectively. *B*, the complex interface can be divided into a major binding site (*solid line square*) and a minor binding site (*dotted line circle*). Contacting residues are represented as *sticks*, while transparent surfaces denote residues involved in van der Waals or packing interactions. Potential hydrogen bonds are represented as *dashed lines*. *C*, detailed view of the interactions in the major site. For clarity, only stacking interactions are shown on the two *left panels* while the hydrogen bonding residues are shown only in the two *rightmost panels*. In these panels, interacting atoms of side chains or main chain atoms are displayed only if they participate in the interactions. *D*, detailed view of the interactions at the minor site. The *gray sphere* denotes the Ca^2+^ ion bound to the Fg domain of TNC. *E*, comparison of the RPTPζ-TNC– and the RPTPζ-TNR–binding sites. The *color* coding for the RPTPζ-TNC complex is identical to the one used in *panel A*, while residues in the RPTPζ–TNR complex are shown in *transparent white*. For clarity, only the relevant main chain or side chain atoms are represented. Fg, fibrinogen-like; FN, fibronectin type III; RPTPζ, protein tyrosine phosphatase receptor type Z; TNC, tenascin-C; TNR, tenascin-R.

Next, it was of interest to determine whether specific differences exist in the interactions between RPTPζ and TNR/TNC. Crystallization trials were thus undertaken with the FN domain of RPTPζ and the Fg domain of TNC mixed in a 1:1 molar ratio. The crystal structure of the RPTPζ–TNC complex was determined and refined to 1.89 Å (R_work_/R_free_ = 0.164/0.205, Table 1, Fig. 5). The complex formed by RPTPζ and TNC is almost indistinguishable from the one formed by RPTPζ and TNR (Fig. 6*A*). The single RPTPζ-TNC found in the asymmetric unit superimposes onto each of the four complexes found in the RPTPζ-TNR crystal unit cell with RMSD values of 0.717 to 1.107 Å (313–316 Cα positions). It occludes a total of 935 Å^2^ with a shape complementarity value of 0.75. These values compare well with those calculated for the RPTPζ–TNR interface. In general, contacts between residues in RPTPζ and in TNC also assort between a major and a minor site (Fig. 6, *B*–*D*). In the major contact site, R334 and Y368 in RPTPζ interlock with H2163, H2175, and R2151 in TNC (Fig. 6*C*), while Q369 forms five hydrogen bonds with S2164, H2150, C2149, and Y2140 (Fig. 6*D*). Furthermore, the Oδ2 atom of D366 forms a hydrogen bond with the amide group of the N2148 side chain. In the minor site located close to the Ca^2+^ ion bound to TNC (Fig. 6*D*), V2133 is nestled against L329 and I374 in RPTPζ, while the carboxylate group of D370 makes hydrogen bonds with the main chain nitrogen atoms of A2132 and V2133. Finally, this carboxylate group also hydrogen bonds with the side chain oxygen atom of T2134. Comparison of the RPTPζ–TNC and RPTPζ–TNR interfaces indicates that most contacts are conserved between the complexes (Fig. 6*E*). However, M342 in RPTPζ does not contact residues in TNC. Furthermore, the hydrogen bond between D366 and TNC residue N2148 (N1302 in TNR) is found in only two of the four RPTPζ–TNR complexes, while the bond between the side chain atoms of D370 in RPTPζ and T2134 in TNC (T1288 in TNR) is present in two of the four RPTPζ–TNR complexes. This contact with T2134, a residue that coordinates the Ca^2+^ ion, might account for the more significant effect that removal of Ca^2+^ has on interactions between TNC and RPTPζ. However, in spite of these differences, these results indicate that RPTPζ interacts with TNC and TNR *via* similar interfaces.

### RPTPζ does not interact with the Fg domains of TNN or TNX

**Figure 7:**
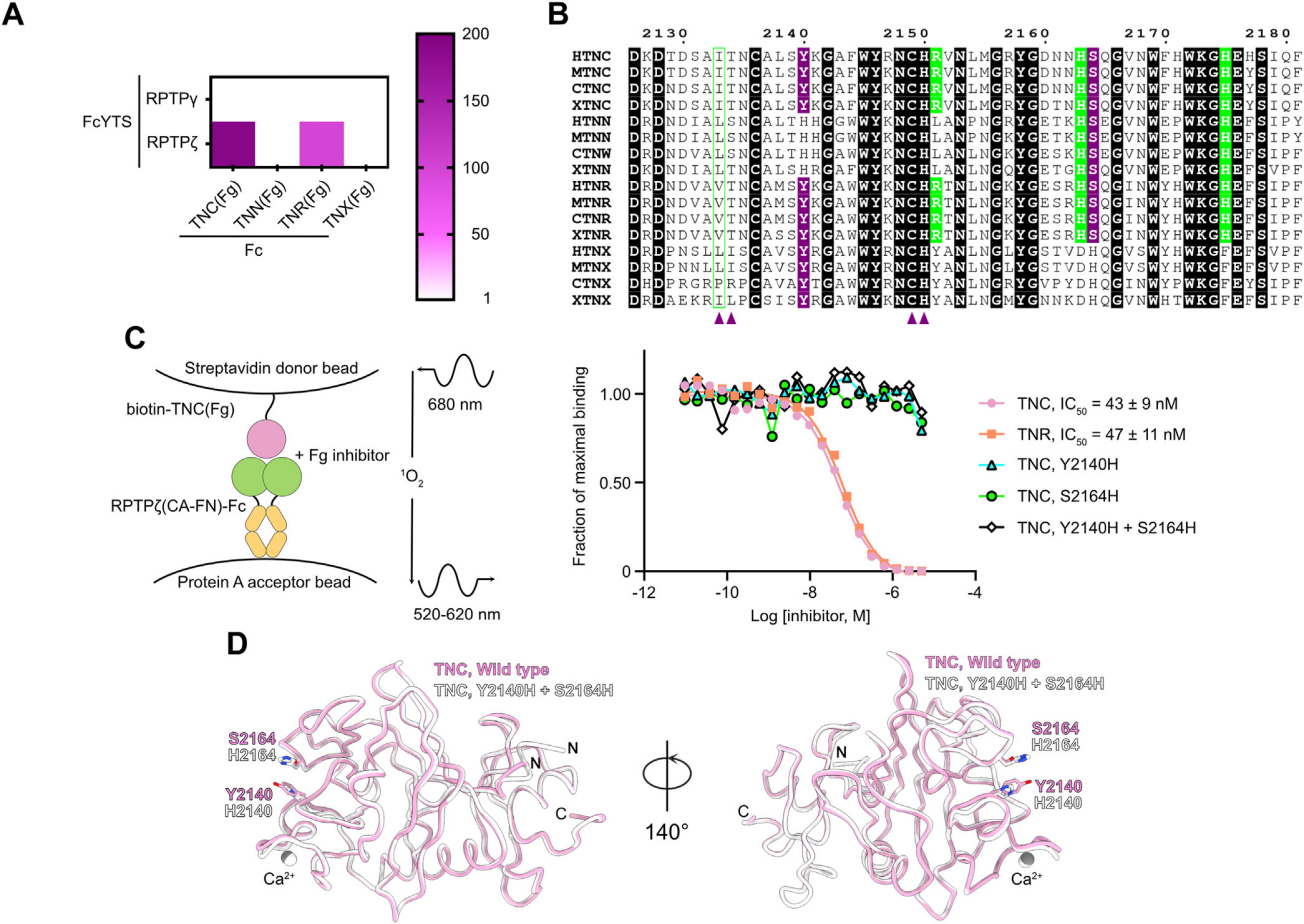
**Two amino acid changes account for the lack of interaction between RPTPζ and TNN or TNX.***A*, results of the binding assay between Fg domains of TNC, TNN, TNR, TNX, and RPTPγ, or RPTPζ are shown in a heat map representation. The scale indicates the value of the signal for the protein pair protein 1-FcYTS/protein 2-Fc divided by the signal measured for the protein 1-FcYTS/Fc only pair. RPTPζ only interacts with TNC and TNR. The assay was performed four times with similar results. One representative experiment is shown. *B*, amino acid conservation at the RPTPζ-binding site in TNC, TNN, TNR, and TNX in human (H), mouse (M), chicken (C), and *Xenopus* (X). An alignment of Fg domains from human, mouse, chicken, and *xenopus* tenascins indicates that amino acid residues at the RPTPζ-binding site are well conserved across family members and across species. Identical residues are shaded in *black*. The side chain atoms of residues shaded *purple* form hydrogen bonds with RPTPζ residues. *Purple triangles* denote residues the main chain atoms of which form hydrogen bonds with RPTPζ residues. Residues involved in stacking interactions are indicated by *green blocks*. Finally, similar residues involved in stacking interactions are shown with a *green box*. The numbering corresponds to human TNC. *C*, mutational analysis of the interactions between the CA-FN region of RPTPζ and the Fg domains of TNC. The ability of TNC(Fg), TNR(Fg), or mutants of TNC(Fg) to inhibit the binding between an IgG Fc fusion of RPTPζ(CA-FN) and a biotinylated TNC(Fg) was assessed over a logarithmic dilution series in an AlphaScreen bead-based competition assay. IC_50_ values are reported as averages ± SDs from at least four experiments. One representative experiment for each series is shown. More detailed information about individual experiments is provided in Table S1. A schematic representation of the assay design is shown on the *left panel*. *D*, the structure of TNC(Fg) determined in the presence of RPTPζ (*pink*) is shown in coil representation overlaid onto the crystal structure of TNC(Fg) in which RPTPζ-binding residues Y2140 and S2164 have both been changed to histidine (*white*). The position of Ca^2+^ ions bound to the Fg domains are indicated by *gray* and *white spheres*, while the letters N and C indicate the N- and C-termini, respectively. The two domains superimpose with a RMSD of 0.34 Å over 216 Cα positions. CA, carbonic anhydrase-like; FN, fibronectin type III; Fg, fibrinogen; IC_50_, half-maximal inhibitory concentration; RMSD, root mean square deviation; RPTPγ, protein tyrosine phosphatase receptor type G; RPTPζ, protein tyrosine phosphatase receptor type Z; TNC, tenascin-C; TNN, tenascin-N; TNR, tenascin-R; TNX, tenascin-X.

In addition to TNC and TNR, the tenascin family includes two additional members: tenascin-N (TNN), which is also known as tenascin-W in chicken, and tenascin-X (TNX) (37). Human TNN and TNX are organized similarly to TNR and TNC. Specifically, they include C-terminal Fg domains that respectively share 56% and 54% amino acid identity the Fg domain of TNR. Thus, we wondered whether the Fg domains of TNN and TNX would interact with RPTPζ. We also wanted to extend this work to RPTPγ, since no binding partner for its single FN domain is known. We expressed the Fg domains of human TNN and TNX as fusion proteins with human IgG1 Fc and assessed their interaction with RPTPζ and RPTPγ in our Alpha-based interaction assay (Fig. 7*A*). These experiments showed that the Fg domains of TNN or TNX do not associate with RPTPζ and that RPTPγ does not bind any of the Fg domains found in the tenascin family.

Using sequence alignments, we identified two amino acid substitutions that could account for the lack of interaction between RPTPζ and TNN or TNX (Fig. 7*B*). First, Y2140 in TNC (Y1294 in TNR), whose side chain hydroxyl group makes a hydrogen bond to the main chain carbonyl group of Q369 in RPTPζ, is conserved in TNX but replaced by a histidine in TNN. Second, S2164 in TNC (S1318 in TNR) is conserved in TNN but replaced by a histidine in TNX. The effect of the Y2140H and S2164H mutations in the interactions between TNC and RPTPζ were thus analyzed in an Alpha-based competition binding assay (38). In these assays, we tested the ability of TNC(Fg), TNR(Fg), and mutants of TNC(Fg) to inhibit the interaction between a Fc fusion of RPTPζ bound to a protein A-acceptor bead and the biotinylated Fg domain of human TNC immobilized on a streptavidin donor bead (Fig. 7*C*). Consistent with the results obtained by BLI, there is little difference in the IC_50_ values measured for the Fg domains of TNC and TNR. These results confirm that RPTPζ interacts with both proteins *via* similar interfaces (Fig. 6*E*). Because our structural analyses strongly suggest that RPTPζ binds to TNC and TNR using similar binding modes, mutations were only introduced in the Fg domain of TNC to investigate the effect of amino acid substitutions found in TNN and TNX. Introducing changes to histidine at positions Y2140 or S2164 prevented competition, suggesting that each mutation is enough to eliminate interaction with RPTPζ (Fig. 7*C*). Not surprisingly, a variant of TNC that includes both the Y2140H and S2164H mutations could not inhibit binding between RPTPζ and WT TNC. Finally, the crystal structure of this variant is identical to that of WT TNC (Table 1 and Fig. 7*D*), indicating that mutations at positions Y2140 or S2164 did not introduce any gross structural alteration that would account for the lack of interaction. Overall, these results validate our structural analyses of the interactions between RPTPζ, TNR, and TNC and explain why RPTPζ does not associate with additional tenascin family members.

### Physical interactions between RPTPζ and TNR are essential for the formation of PNNs

**Figure 8:**
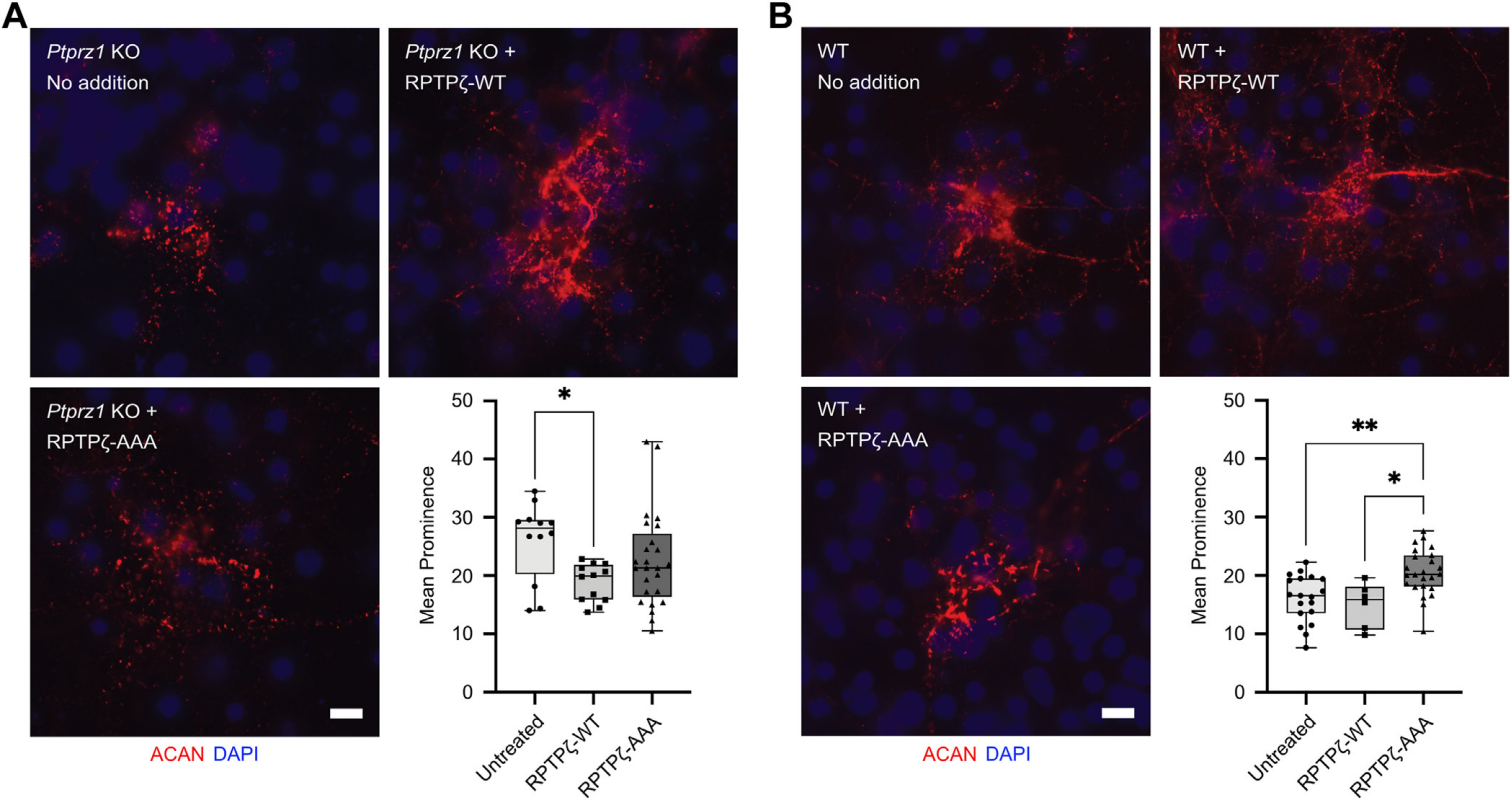
**The assembly of PNNs depends on interactions between RPTPζ and TNR.***A*, addition of RPTPζ-AAA mutant is unable to recover binding of PNN component ACAN in *Ptprz1* KO neuronal cultures. Cortical cultures from E16 *Ptprz1* KO mice were fixed at DIV 9. Binding of PNN component ACAN appeared disrupted with a broken and aggregated staining pattern in these cultures. Addition of recombinant RPTPζ-WT (2 μg per well at DIV 3) to the cells was able to restore regular pattern of aggrecan binding to the cell surface. However, TNR-binding mutant RPTPζ-AAA was unable to restore ACAN binding to the cell. Similar to untreated cultures, the ACAN staining pattern remained broken and discontinuous with an aggregated and punctate appearance. Mean prominence or isolation of PNN peaks was visualized using PNN marker ACAN and quantified using our PNN node/peak analysis. Analysis by ordinary one-way ANOVA showed that the average isolation or prominence of PNN peaks is significantly different between groups (F(2,46) = 3.232, *p* = 0.048). RPTPζ-WT–treated cells (n = 12 cells, 3 cultures) showed a significantly lower mean prominence (∗*p* = 0.037, Tukey’s post hoc testing) than untreated cells (n = 12 cells, 3 cultures) indicating more regular pattern of ACAN staining and a decrease in node/peak isolation. Mean prominence of peaks in RPTPζ-AAA–treated PNNs (n = 25 cells, 3 cultures) was not significantly different from untreated cells (RPTPζ-AAA *p* = 0.35, Tukey’s post hoc testing). *B*, addition of RPTPζ-AAA disrupts binding of PNN component ACAN in WT neuronal cultures. Cortical cultures derived from E16 WT mice were fixed at DIV 9 and stained with PNN marker, ACAN. ACAN staining appeared regular and continuous in untreated group. Addition of RPTPζ-AAA (2 μg per well at DIV 6) resulted in disrupted and aggregated aggrecan staining similar to pattern seen in *Ptprz1* KO cultures. Interestingly, addition of RPTPζ-WT (2 μg per well at DIV 6) did not result in disruption of ACAN binding to the cell surface. PNNs were visualized using PNN marker ACAN, and their mean prominence or isolation was calculated using our PNN node/peak analysis. There were significant differences in the average isolation index of PNN peaks among the various treatment groups (F(2,66) = 6.203, *p* = 0.0034, ordinary one-way ANOVA). Average isolation index or mean prominence of PNNs was significantly higher in RPTPζ-AAA–treated cells (n = 24 PNNs, 6 cultures) as compared to untreated control cells (n = 31 PNNs, 6 cultures, ∗*p* = 0.0079) and RPTPζ-WT–treated cells (n = 14 PNNs, 4 cultures, #, *p* = 0.013). Scale bar represents 10 μm. ACAN, aggrecan; ANOVA, analysis of variance; DIV, day *in vitro*; E, embryonic day; PNN, perineuronal net; RPTPζ, protein tyrosine phosphatase receptor type Z; TNR, tenascin-R.

We next wanted to leverage our structural insights between RPTPζ and TNR to assess the extent to which the complex they form participates in the assembly of PNNs. We recently developed an imaging technique to quantify the presence of PNNs in dissociated mouse cortical cultures (15). In normal nets, the PNN marker ACAN is distributed in a regular, lattice-like structure at the neuronal surface (9). In contrast, PNNs are distributed discontinuously on the surface of neurons harvested from mice lacking *Ptprz1* (15), which leads to the formation of prominent foci of ACAN (Fig. 8*A*, *No addition*). We can thus investigate the formation of PNNs in cultured neurons by quantifying the number and prominence of these nodes.

Because it was suspected that formation of the RPTPζ–TNR complex identified in our crystals plays a role in the assembly of PNNs, we reasoned that a mutant form of RPTPζ that does not bind to TNR would not be able to rescue the formation of PNNs in dissociated neurons lacking *Ptprz1*. First, we tested the ability of a fusion protein between amino acids 34–629 of the RPTPζ ectodomain and human IgG1 Fc to rescue PNN structure in dissociated primary neurons isolated from *Ptprz1*^−/−^ mice. In these experiments, exogenous addition of WT RPTPζ-Fc recovered the PNN structures (Fig. 8*A*, *RPTPζ-WT*). The extent of the recovery compared well with what was previously observed when *Ptprz1*^−/−^-dissociated neurons were treated with phosphacan (15). This finding suggests that the large GAG insert region found at the C-terminus of phosphacan does not appear to play a significant role in the formation of PNNs (13). Next, we introduced alanine residues in place of amino acids Y368, Q369, and D370 in the RPTPζ-Fc fusion protein to produce a form of RPTPζ, designated RPTPζ-AAA, that does not interact with TNR. In this case, treatment with the RPTPζ-AAA did not rescue the discontinuous appearance of nets in cultured *Ptprz1*^−/−^ neurons (Fig. 8*A*, *RPTPζ-AAA*). In complementary experiments, we investigated if addition of RPTPζ-AAA could alter PNN structures in WT neurons (Fig. 8*B*). Whereas addition of RPTPζ-Fc has no significant effect on WT PNN structures formed in dissociated cultures (Fig. 8*B*, *No addition* & *RPTPζ-WT*), addition of RPTPζ-AAA caused PNN assemblies to disaggregate and take on a disorganized appearance similar to structures found in cortical cultures of *Ptprz1*^−/−^ neurons. Taken together, these experiments strongly support the idea that interactions between RPTPζ and TNR are essential for the assembly of PNNs.

## Discussion

PNNs are considered essential to ushering the end of the hyperplastic period necessary for the proper development of the CNS, and yet the processes that lead to their assembly have not been explained fully. In particular, the identities of the proteins that are critical to forming PNNs remain understudied. These include secreted proteins that associate with known net components such as TNR and lecticans as well as cell surface receptors that can anchor PNNs to neuronal surfaces. In that context, the discovery that the loss of the proteoglycan phosphacan impaired formation of the reticular PNN structure provided a novel opportunity to dissect the anatomy of PNNs (15).

In the current studies, we have exploited the RPTPζ KO model to demonstrate that the assembly of PNNs depends on the formation of a complex between RPTPζ/phosphacan and the matricellular protein TNR. Importantly, we showed that a phosphacan construct lacking the GAG attachment region rescues PNN structure. This result suggests that the GAG chains found on phosphacan do not appear essential for its role in PNN formation. Subsequently, we determined from the cocrystal structure of RPTPζ and TNR that a loop in the single FN domain found in phosphacan inserts inside a groove in the C-terminal Fg domain of TNR and that mutations of amino acid residues in this loop disrupts formation of PNNs. Utilizing this information, we designed a construct of RPTPζ that does not bind TNR and proved that the interaction with TNR is critical for PNN structure. To the best of our knowledge, these results also provide the first structural insights into a complex formed by the single FN domain found in RPTPζ/phosphacan.

Although the combined data presented here and in a previously published report establish that phosphacan–TNR interactions are essential to maintain PNN assembly (15), the function that phosphacan might have in the architecture of nets is still an open question. Even more puzzling, the fact that adding a soluble protein such as phosphacan rescues the formation of PNNs in *Ptprz1*-null mice suggests that the transmembrane form of phosphacan, RPTPζ, is not necessary for PNN formation. These findings are reminiscent of the function that phosphacan plays in the maturation of oligodendrocyte precursor cells (OPCs): genetic removal of *Ptprz1* impaired OPC differentiation and maturation, but the phenotype could be rescued by addition of phosphacan (27). Which role could phosphacan fulfill in the context of nets? There are two, nonmutually exclusive, possibilities. First, phosphacan has been reported to bind to neurocan (39), a member of the lectican family that has been identified in PNNs (40, 41). Thus, phosphacan could crosslink net structures by linking TNR and neurocan. Second, phosphacan associates with multiple cell surface receptors including CNTN1, L1, and NCAM (42, 43). In this case, phosphacan might help anchor nets to the neuronal cell surface. Future work will be required to disentangle these two possibilities and address the function of phosphacan in PNNs.

The results reported here also indicate that RPTPζ binds to TNC in almost identical fashion to TNR. TNC is found in PNNs, and plasticity is altered in mice lacking *Tnc* (20). However, the formation of nets is not impaired in *Tnc*^−/−^ mice, so that TNC does not appear essential to the assembly of PNNs (19). In that context, what could be a physiological function for the RPTPζ–TNC complex? The first possibility is that even though TNC is not essential in the formation of PNNs, it plays a hitherto unidentified role in their plasticity-restricting function that requires interaction with RPTPζ. Another possibility is that the physiological functions of RPTPζ–TNC complexes are not related to PNNs. In that context, it would be interesting to examine the role of RPTPζ and TNC in the maturation of OPCs and myelination. Indeed, both proteins have been linked to these processes although there are inconsistencies about the exact role of RPTPζ in the development of OPCs (27, 44, 45). That being said, examining a potential role of RPTPζ and TNC in the development and function of oligodendrocytes should be considered along with TNR, which appears to function similarly to TNC in this process (46). Finally, it will be of interest to consider the possibility that TNC might bind to the transmembrane form of RPTPζ and the effect that it might have on the phosphatase activity of the receptor. This could be especially relevant in the context of neural stem cells such as outer radial glia, which appears to express both RPTPζ and TNC (47). The structural results presented here along with the identification of point mutations in RPTPζ and TNC that impair the formation of complexes between RPTPζ and TNC or TNR should help the design of experiments aimed at providing answers to these critical questions.

## Experimental procedures

### Cloning

A complementary DNA (cDNA) construct encoding the extracellular region of human CNTN1 excluding the GPI anchor region (amino acids 1–991) was synthesized by Genscript. cDNA constructs encoding amino acid residues 34 to 629 of human RPTPζ, a related construct in which Y368, Q369, and D370 are replaced by alanine, and amino acids residues 54 to 728 of mouse RPTPγ were generated by PCR. These CNTN1, RPTPζ, and RPTPγ constructs were cloned into derivatives of the pLex2 vector to express these proteins as fusions with human IgG1 Fc and chicken IgY Fc fused to a Twin-Strep tag (25). PCR was also used to generate cDNA fragments encoding the carbonic anhydrase-like (CA, amino acid 34–302), FN (amino acid residues 310–410), and CA-FN (amino acid residues 34–410) domains of human RPTPζ. These fragments were also ligated in the pLex2 vector to express the corresponding proteins as fusions with human IgG1 Fc.

Attempts to express tenascin fragments including the Fg domains as an N-terminal fusion with human IgG1 Fc were unsuccessful, so a different strategy was designed. cDNA fragments corresponding to regions of tenascin family members were obtained from Integrated DNA Technologies or Genscript and cloned into a derivative of the pLex2 vector at the 3′ end of the sequence encoding the Fc region of human IgG1 so that proteins of interest are fused to the C-terminus of the Fc domain. A similar expression vector directing the expression of tenascin fragments C-terminally to a Twin-Strep tag followed by Cν2 and Cν3 domains of chicken IgY was designed. In the case of human TNC, these fragments encode amino acid residues 187 to 621 (EGF domains), 622-1071+1709-2201 (FN1-Fg domains), and 1072-1253+1345-1526+1618-1708 (domains A1A2A4BD). In the case of human TNR, the cDNA fragments encode amino acid residues 201 to 324 and residues 325 to 1358 corresponding to the EGF and FN1-Fg regions, respectively. These N-terminal human IgG1 Fc and chicken IgY Fc expression vectors were finally employed to express an ACAN construct from which the GAG regions were deleted. This cDNA encodes amino acid residues 31 to 2530 of ACAN in which residues 680 to 2276 are replaced by the dipeptide GS. It was synthesized *de novo* by Synbio Technologies. Finally, we engineered a derivative of pLex2 that includes an Emerald fluorescent tag after the signal sequence in the vector (48, 49, 50). cDNA fragments encoding the ectodomains of human RPTPζ isoform 3 (amino acid residues 34–774) and mouse RPTPγ (amino acid residues 54–728) without their respective signal sequence were then inserted after the Emerald sequence to express RPTPζ and RPTPγ with a fluorescent tag at their respective N-termini.

cDNA fragments encoding the FN domain of human RPTPζ (amino acid residues 280–381) and the Fg domains of human TNC (amino acid residues 1975–2201) and TNR (amino acid residues 1129–1358) were optimized for expression in bacteria, designed with BamHI and EcoRI restriction sites at their 5′ and 3′ ends, respectively, and purchased from Integrated DNA Technologies. These fragments were digested with the restrictions enzymes BamHI and EcoRI and cloned into a derivative of the pET32 plasmid that includes human rhinovirus 3C protease site between the hexhistidine sequence and the protein of interest (38). Plasmids that direct the expression of variants of TNC(Fg) that include the S2140H and Y2164H changes were produced similarly. All plasmid constructs were verified by DNA sequencing.

### Protein expression and purification

The FN domain of human RPTPζ and the Fg domains of human TNR as well as WT and mutant forms of TNC were expressed in *Escherichia coli* strain Origami 2(DE3). Overnight cultures–transformed bacteria (100 ml) were diluted into 4 L of Luria-Bertani broth containing 50 μg/ml of carbenicillin, and the cultures were grown at 30 °C and 225 rpm. Protein expression was induced when A600 reached 0.8 by adding IPTG to a final concentration of 0.5 mM. The temperature was decreased to 19 °C, and cells were grown for a further 16 to 18 h. Harvested cells were suspended in 50 ml of loading buffer (500 mM NaCl, 25 mM imidazole, and 50 mM sodium phosphate, pH 7.5) and lysed by microfluidization. The lysate was centrifuged 30 min at 20,000*g*, and the supernatant was applied to a 4-mL column of Ni Sepharose 6 Fast Flow (Cytiva) equilibrated in lysis buffer chelating sepharose. The column was washed with loading buffer, and bound proteins were eluted with loading buffer containing 250 mM imidazole. After cleavage with human rhinovirus 3C protease and dialysis against loading buffer, the proteins were passed over a 5-mL His-Trap column (Cytiva) and the flow through was kept. Subsequent purification steps involved ion exchange on a 5-mL HiTrap Q HP column (Cytiva) equilibrated in 20 mM Tris–HCl pH 8.0 (RPTPζ) or affinity chromatography on a 5-mL HiTrap Heparin column (Cytiva) equilibrated in 40 mM sodium phosphate pH 7.5 (TNC and TNR) followed by gel filtration chromatography on a Superdex 200 26/600 column (Cytiva) equilibrated in 150 mM NaCl and 20 mM Na–Hepes pH 7.5.

For biotinylation, human RPTPζ(FN) in gel filtration buffer (150 mM NaCl, 20 mM Na–Hepes pH 7.5) was incubated with a 1:1 molar ratio of EZ-Link NHS-PEG4-Biotin (Thermo Fisher Scientific) for 2 h on ice according to the manufacturer’s instructions. To avoid problems linked to aggregation following concentration in centrifugal devices, the most concentrated fraction from the size-exclusion purification was selected for biotinylation. The reaction was quenched by adding Tris–HCl pH 8.0 to a final concentration of 100 mM, and the protein was extensively dialyzed at 4 °C against 150 mM NaCl and 20 mM Na–Hepes pH 7.5. To generate biotinylated human TNC(Fg), a cysteine residue was added at the N-terminus of the Fg domain by site-directed mutagenesis. The purified protein was then incubated with EZ-Link Maleimide-PEG2-Biotin (Thermo Fisher Scientific) according to the manufacturer’s instructions. Biotinylated proteins were subsequently purified by gel-filtration chromatography as described above. Proteins were then stored at 4 °C until use in binding assays or crystallization trials.

Proteins used in Alpha-based binding assays were expressed in HEK 293T/17 cells (ATCC # CRL-11268) that were maintained in Dulbecco’s modified Eagle’s medium high glucose supplemented with 10% (v/v) of fetal bovine serum (FBS), supplemented with antibiotics and nonessential amino acids. Plasmids encoding Fc or FcYTS fusion proteins were transfected transiently in cells plated in 6-well dishes using PEI (51). Cells were maintained in Opti-MEM™ media (Thermo Fisher Scientific) supplemented with 1% (v/v) of ultra-low IgG FBS (Thermo Fisher Scientific) and nonessential amino acids after transfection. After 3 to 5 days, conditioned media were harvested and dialyzed against three changes of 150 mM NaCl, 20 mM Na–Hepes pH 7.5 to remove traces of biotin prior to the experiments. Proteins could be used immediately or aliquoted and placed at −80 °C for long-term storage. For the competition Alpha-binding assays, the CA and FN domains of human RPTPζ were expressed as human IgG1 Fc fusion proteins using transient transfection in HEK 293T/17 cells grown in three 150 cm^2^ round dishes. Conditioned media (∼100 ml) was harvested after 5 days, and the protein was purified by affinity chromatography using a 1-mL HiTrap Protein A column (Cytiva) according to the manufacturer’s instructions. The eluted protein was more than 95% pure as judged by SDS PAGE. It was then dialyzed against 150 mM NaCl and 20 mM Na–Hepes pH 7.5 and stored.

### Alphascreen protein-binding assays

The interactions between ACAN, CNTN1, RPTPγ, RPTPζ, and tenascin family members were analyzed using an extracellular binding assay (24). Candidate proteins were fused with either human IgG1 Fc or domains Cν3-Cν4 of chicken IgY tagged with a Twin-Strep peptide and expressed in HEK293 cells as described above. To run the assay, aliquots (7.5 μl) of candidate proteins fused to Fc were pipetted into an 96-well plate followed by addition of an equal volume of candidate proteins fused to FcYTS. A solution of Strep-Tactin Alpha donor beads (PerkinElmer Life Sciences, 37.5 μg/ml) and AlphaScreen Protein A acceptor beads (PerkinElmer Life Sciences, 37.5 μg/ml) were mixed in 150 mM NaCl, 20 mM Na–Hepes pH 7.5, 2 mM CaCl_2_, 2 mM MgCl_2_, 2 mg/ml bovine serum albumin, 0.2% (v/v) Triton-X100, and 0.2% (v/v) Tween-20. Aliquots (15 μl) of this solution were immediately added to the wells containing the candidate proteins. The well contents were then transferred to a 96-well ½ area opaque microplates. After a one-hour incubation at room temperature, plates were analyzed on an EnSpire multimode plate reader (PerkinElmer Life Sciences). For each candidate protein, the intensities were normalized by dividing the measured luminescence signal by the signal obtained for the negative control that includes the candidate protein fused to the FcYTS tag and an Fc-only sample. Experiments were then repeated with the tag swapped between the two candidate proteins (for example CNTN1-Fc *versus* RPTPζ-FcYTS and CNTN1-FcYTS *versus* RPTPζ-Fc). We defined an interaction pair as one for which we measure a signal over background of at least two for each orientation (protein 1-Fc/protein 2-FcYTS and protein 2-Fc/protein 1-FcYTS). Protein pairs for which luminescence signals were identified in only one orientation were ignored. Experiments performed only in a single orientation were carried out at least twice. Raw signals for these assays are included as supporting information (Table S1).

AlphaScreen binding assays were also used in a competition format to characterize the interactions between RPTPζ and mutants of TNC. The beads used in the assay were obtained from an AlphaScreen general IgG (Protein A) detection kit. Reactions (25 μl final volume) were initially set up in sealed 96-well microplates to prevent evaporation. Assays were initiated by mixing 5 μl of biotinylated human TNC(Fg) (5 nM final concentration) with 5 μl of purified human RPTPζ(CA-FN) expressed as human IgG1 Fc fusion proteins in each well (0.5 nM final concentration). Aliquots (5 μl) of untagged, WT and mutant TNC(Fg) of varying concentrations were added to the reactions. Protein A-coated acceptor beads (5 μl, 20 μg/ml final concentration) were then added to each well. After a 1-h incubation at room temperature, streptavidin-coated donor beads (5 μl, 20 μg/ml final concentration) were added to each well. The reactions were allowed to stand at room temperature for 30 min prior to transfer to 96-well ½ area opaque microplates for detection using an EnSpire multimode plate reader (PerkinElmer Life Sciences). Values for normalized binding were calculated by dividing the signal measured for a reaction without inhibitor. Results were fitted to a one-site competition equation, in which the IC_50_ is the concentration of inhibitor that gives 50% inhibition of maximal binding using Prism 8 (GraphPad Software; https://www.graphpad.com). The values of IC_50_ are reported as averages ± SDs from at least four experiments (Table S1). No attempt was made to fit curves for the TNC mutants because they did not inhibit the interaction between WT TNC and RPTPζ.

### BLI experiments

Interactions between the FN domain of RPTPζ and the Fg domains of human TNR or TNC were quantified at room temperature in 150 mM NaCl, 10 mM Na–Hepes pH 7.5, 1 mg/ml bovine serum albumin, 0.02% (v/v) Tween-20 using an Octet K2 system (Sartorius) in the presence of either 5 mM CaCl_2_ or 5 mM EDTA. The biotinylated FN domain of RPTPζ (250 nM) was immobilized onto streptavidin tips (Sartorius). These tips were then incubated with purified TNC(Fg) or TNR(Fg) at a series of concentrations for 90 s during the association phase, by which time the signal had reach a plateau. The tips were then incubated in buffer only during the dissociation phase for 90 s. The signal was corrected by subtracting the background measured for the buffer only. In the experiments reported here, the observed dissociation rate constants attained or exceeded the accuracy limits of the K2 instrument (koff > 0.01 s^−1^, Table S1). Thus, the dissociation constants (K_D_) were calculated by plotting the values of the maximal binding signal obtained at equilibrium against the concentration of Fg domains and fit using Prism 9 (GraphPad Software) to the equation: signal = max ∗ C/(K_D_ + C) where max is the normalized maximal-binding signal and C is the concentration of Fg domain. The results are reported as the average of at least four replicates.

### Cell surface binding assays

The FN1-Fg or Fg domains of human TNR and TNC were expressed transiently in HEK 293T/17 cells as described above. After 3 days, conditioned media were harvested and stored at 4 °C. Prior to cell staining, 1 ml of conditioned media was supplemented with 2% (v/v) of normal goat serum and 1 μg of goat anti-human IgG derivatized with Alexa Fluor 568 (Thermo Fisher Scientific) and incubated on ice for 30 min. Plasmids encoding the Emerald-tagged versions of RPTPγ and RPTPζ were transfected transiently in HEK 293T/17 cells grown on coverslips placed in 12-well dishes. Two days after transfection, the media was removed and the cells were washed three times with PBS and incubated with 1 ml of conditioned media containing TNC/TNR and goat anti-human IgG Alexa Fluor 568. After incubation at room temperature, the cells were washed three times with PBS and then fixed in 4% (w/v) paraformaldehyde in PBS for 10 min. All images were acquired sequentially using a Zeiss LSM 510 confocal microscope with appropriate lasers for excitation (488 and 561 nm) and filters (505-530BP and 565-600BP, respectively). Images were processed for publication using Fiji (https://fiji.sc) (52). For quantification of binding of TNR and TNC to transfected cell surfaces, average intensity of TNR and TNC in the images was measured using the measure function in Fiji. Background correction was carried out by choosing three separate areas devoid of cellular staining (100px × 100px) and subtracting the average intensity in these regions from the cell surface intensity of TNR/TNC staining. Graphs are represented as fold change normalized to appropriate controls. Analyses were conducted on images derived from three independent experiments.

### Crystallization, structure determination, and structural analyses

Unless indicated otherwise, crystallization trials were initiated by mixing 1 μl aliquots of protein or complex with 1 μl of mother liquor prior to crystallization. All crystals were grown then at 20 °C by hanging drop vapor diffusion. For crystallization of the RPTPζ–TNR complex, purified RPTPζ(FN) was mixed with TNR(Fg) in a 1:1 molar ratio at a final concentration of 100 μM in 5 mM CaCl_2_, 5 mM Na–Hepes pH 7.5, and 40 mM NaCl. The complex solution (2 μl) was then mixed with 1 μl of 20% (w/v) PEG 2000 MME, 200 mM KSCN. Crystals were frozen in mother liquor supplemented with 10% (v/v) glycerol. The RPTPζ–TNC complex was reconstituted by mixing purified RPTPζ(FN) and TNC(Fg) in a 1:1 molar ratio at a final concentration of 275 μM in 5 mM CaCl_2_, 10 mM Na–Hepes pH 7.5, and 75 mM NaCl. This complex was crystallized in 20% (w/v) PEG 3,350, 100 mM Tris–HCl pH 8.0, and crystals were frozen in 20% (w/v) PEG 3,350, 100 mM Tris–HCl pH 8.0, and 10% (v/v) glycerol. Crystals of the Fg domain of human TNR (300 μM in 2 mM Tris–HCl pH 8.0, 20 mM NaCl, and 5 mM CaCl_2_) appeared in 10% (w/v) PEG 3,350, 200 mM L-Proline, 100 mM Na–Hepes 7.5, and 5 mM CaCl_2_. They were frozen in the same condition supplemented with 25% (v/v) glycerol. The Fg domain of human TNC harboring the Y2140H and S2164H mutations (300 μM in 2 mM Tris–HCl pH 8.0, 20 mM NaCl, and 5 mM CaCl_2_) was crystallized in 25% (w/v) PEG 1,500, 200 mM Li_2_SO_4_, 50 mM Tris–HCl pH 8.0 and frozen in 25% (w/v) PEG 1,500, 100 mM Li_2_SO_4_, 50 mM Tris–HCl pH 8.0, 10% (v/v) glycerol, and 1 mM CaCl_2_.

X-ray diffraction data were collected on beamlines 22-ID and 22-BM of the Advanced Photon Source at Argonne National Laboratory. Diffraction data were processed using HKL2000 (53). Ramachandran and geometry statistics for all models were validated using the RSCB Protein Data Bank validation server. Structures were determined by molecular replacement in PHASER as implemented by PHENIX (54, 55) using the crystal structure of the Fg domain of tachylectin 5A and the crystal of the FN5 domain of TNC as search models (32, 33). The final models were obtained after several rounds of manual rebuilding in COOT (56) and refinement in PHENIX. These models were validated using the RSCB PDB validation server. Shape complementarity coefficients were calculated using SC (34) as implemented by CCP4 (57), while lists of interacting residues and interface areas were obtained using the PISA server (58). Structural representations were generated using ChimeraX (59).

### Animals

Mice lacking the *Ptprz1* gene (*Ptprz1* KO) were generated as described previously (60) and received from Dr Sheila Harroch (Department of Neuroscience, Institut Pasteur). For primary cortical neuronal cultures, in addition to the above *Ptprz1* KO mice, timed pregnant CD-1 WT mice were purchased from Charles River Laboratories. All experiments followed the protocols approved by the Institutional Animal Care and Use Committee of SUNY Upstate Medical University.

### Primary cortical cultures

Primary cortical neuronal cultures were prepared as described previously (12, 61). Briefly, cortices from embryonic day (E) 16 CD-1 WT or *Ptprz1* KO embryos were dissected out and digested with 0.25% trypsin-EDTA (Thermo Fisher Scientific) for 25 min. The tissue ball after trypsin digestion was treated with RNAase-free DNAase (Promega) for 6 min and passed through a 70 μm cell strainer (Falcon). Cells were centrifuged to remove any residual DNAase and resuspended in Neurobasal medium supplemented with B27, GlutaMAX, and penicillin-streptomycin (Thermo Fisher Scientific). Cultures were plated at a density of 2.1 × 10^6^ cells/ml on glass coverslips precoated with poly-D-lysine (100 μg/ml, Sigma-Aldrich) and laminin (50 μg/ml, Thermo Fisher Scientific). Cells were treated with 5 μM cytosine arabinoside (AraC, Sigma-Aldrich) from 1 to 3 days *in vitro* (DIV) to eliminate glia. Culture media was replaced at 3 DIV after AraC treatment, followed by a half media change at 6 DIV.

For RPTPζ-WT and RPTPζ-AAA addition experiments, respective constructs were transfected into HEK293 cells using a PEI transfection method as described above. The transfected cells were switched to serum-free Opti-MEM media (Thermo Fisher Scientific) after 24 h. Conditioned media from transfected HEK293 cells were collected after an additional 24 h and concentrated using a 100,000 MWCO concentrators (AmiconUltra, EMD Millipore). As before, presence of RPTPζ-WT and RPTPζ-AAA in concentrated fractions was verified using dot blots, and total protein was estimated using a Bradford assay (15). A total of 2 μg of protein was added to *Ptprz1* KO and CD-1 cultures at 3 DIV and 6 DIV, respectively. Cells were maintained at 37 °C and 5% CO_2_ until fixation at 9 DIV.

### Immunocytochemistry and immunohistochemistry

Primary cortical cultures plated on coverslips were fixed in cold 4% phosphate-buffered paraformaldehyde with 0.01% glutaraldehyde, pH 7.4 at 9 DIV. Afterward, the cells were blocked in screening medium (Dulbecco’s modified Eagle’s medium, 10% (v/v) FBS, 0.2% (w/v) sodium azide) for 1 h, before adding primary antibodies overnight at 4 °C (rabbit anti-ACAN: millipore-sigma AB1031, mouse anti-TNR: R&D systems MAB1624). The next day, Alexa Fluor–conjugated secondary antibodies (Thermo Fisher Scientific) in screening medium were added to the cells for 2 h before mounting the coverslips with ProLong Antifade Kit (Thermo Fisher Scientific). Cell nuclei were visualized with Hoechst solution (Thermo Fisher Scientific) diluted in PBS.

### Quantification of PNN formation

PNN peak or node analysis was used to quantitatively describe the broken or discontinuous distribution of PNN marker ACAN on the surface of cultured neurons as described previously (15, 61). In short, images of ACAN-positive neurons were processed using the local maxima function of ImageJ to identify peaks (nodes) of intense PNN staining. An ad hoc algorithm was then used to measure the average distance between those nodes and the difference in intensity between the nodes and their surrounding space on the cell surface (node prominence). The number of unique nodes and their mean prominence was plotted for each genotype.

## Data availability

The atomic coordinates and structure factors (codes 8FN8, 8FN9, 8FNA, and 8FNB) have been deposited in the Protein Data Bank (https://www.rcsb.org). All other data are contained within the article and Supporting Information.

## Supporting information

This article contains supporting information (32).

## Conflict of interest

The authors declare that they have no conflict of interest with the contents of this article.
