# Supplementary material for: Protein–protein interactions between tenascin-R and RPTPζ/phosphacan are critical to maintain the architecture of perineuronal nets
Source: J Biol Chem. 2023 Jun 23;299(8):104952. doi: 10.1016/j.jbc.2023.104952 (PMC10371798; doi:10.1016/j.jbc.2023.104952)
Supplement: Supporting Table S1 and Figures S1–S3 [file mmc1.docx]

Supporting information for:

Protein-protein interactions between tenascin-R and RPTPζ/phosphacan are critical to maintain the architecture of perineuronal nets

Ashis Sinha^1^, Jessica Kawakami^2^, Kimberly S. Cole^2^, Aliona Ladutska^2^, Mary Y. Nguyen^2^, Mary S. Zalmai^2^, Brandon L. Holder^2^, Victor M. Broerman^2^, Russell T. Matthews^1,*^, Samuel Bouyain^2,*^

From the ^1^Department of Neuroscience and Physiology, State University of New York Upstate Medical University, Syracuse, New York 13210 and the ^2^Division of Biological and Biomedical Systems, School of Science and Engineering, University of Missouri-Kansas City, Kansas City, Missouri 64110

Corresponding authors: Russell T. Matthews, [matthewr@upstate.edu](mailto:matthewr@upstate.edu); Samuel Bouyain, [bouyains@umkc.edu](mailto:bouyains@umkc.edu).

Supporting information includes:

- Table S1 – Excel spreadsheet, available separately
- Fig. S1, included in this file
- Fig. S2, included in this file
- Fig. S3, included in this file


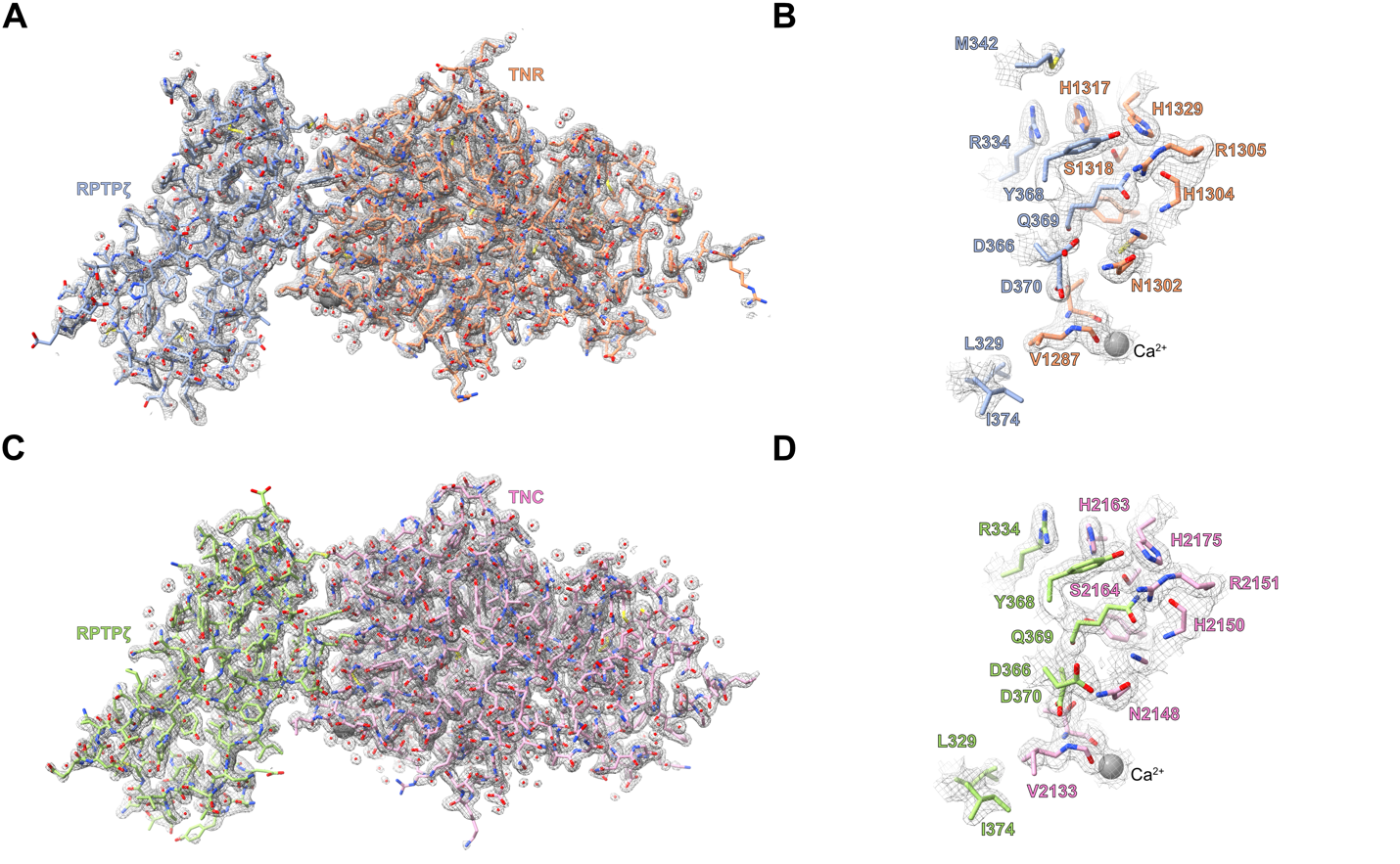


**Fig. S1. Representative electron density maps at the interface between RPTPζ and TNR or TNC.**

1. The RPTPζ-TNR complex is shown here in stick representation along with a 2mFo-DFc electron density map contoured at 1σ.
2. Amino acid residues at the interface between RPTPζ and TNR are shown as sticks along with a 2mFo-DFc electron density map contoured at 1σ.
3. The RPTPζ-TNC complex is shown here in stick representation along with a 2mFo-DFc electron density map contoured at 1σ.
4. Amino acid residues at the interface between RPTPζ and TNC are shown as sticks along with a 2mFo-DFc electron density map contoured at 1σ.

TNC, tenascin-C; TNR, tenascin-R; RPTPζ, protein tyrosine phosphatase receptor type Z


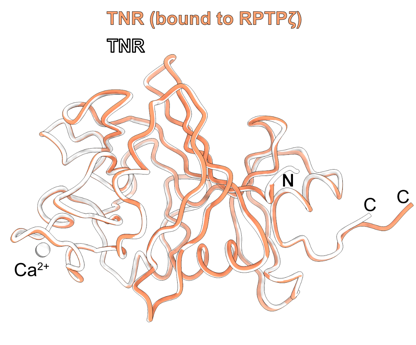


**Fig. S2. Overlay of the Fg domains of TNR in the absence and presence of bound RPTPζ.**

The structure of TNR(Fg) determined in the presence of RPTPζ (salmon) is shown in coil representation overlaid onto the structure of TNR(Fg) determined in the absence of RPTPζ (white). The position of Ca^2+^ ions bound to the Fg domains are indicated by grey and white spheres while the letters N and C indicate the N- and C-termini, respectively. Fg, fibrinogen; TNR, tenascin-R; RPTPζ, protein tyrosine phosphatase receptor type Z


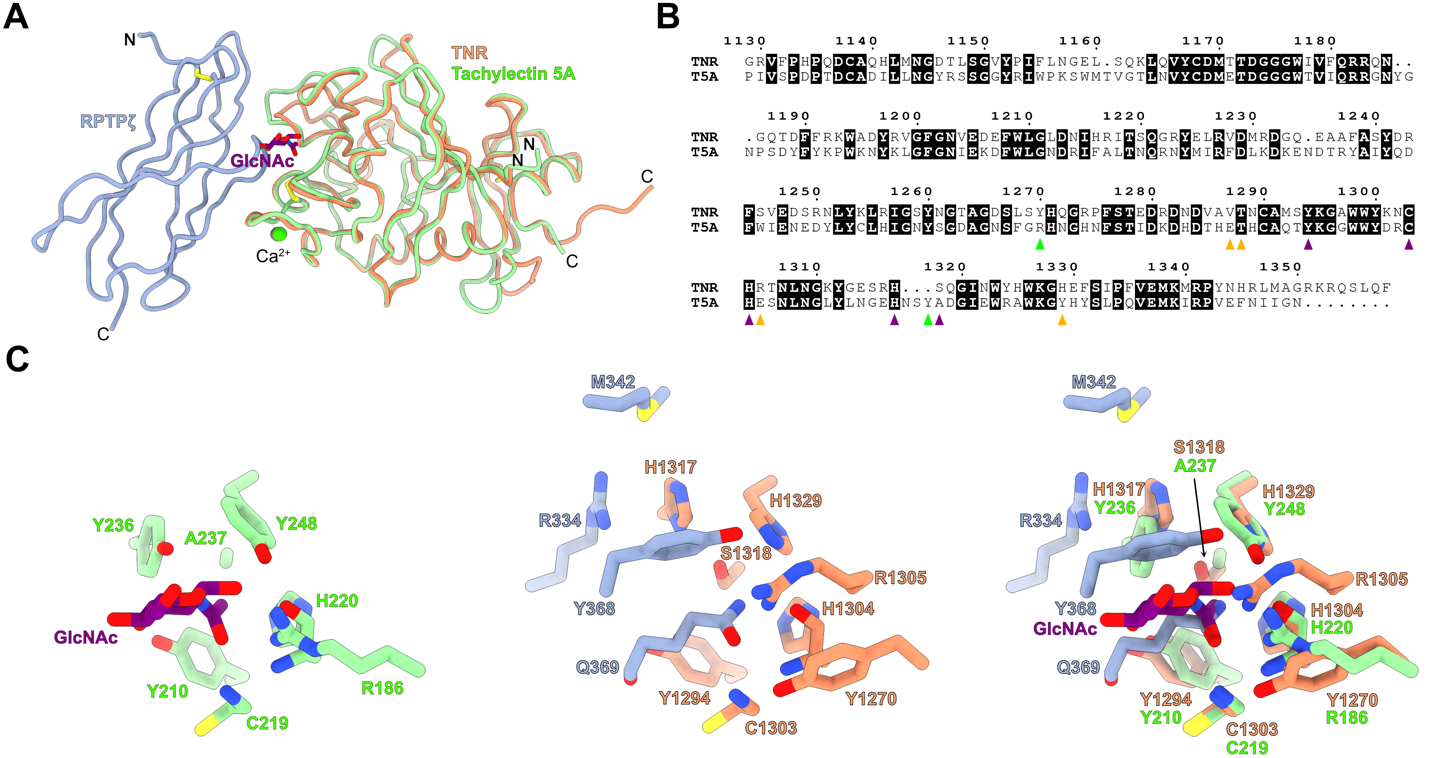


**Fig. S3. The RPTPζ-binding site in TNR(Fg) overlaps with the carbohydrate-binding site in tachylectin 5A.**

1. The RPTPζ-TNR complex is shown as overlaid onto the Fg domain of tachylectin 5A (PDB ID 1JC9). The FN domain of RPTPζ is colored slate, while the Fg domain of TNR is shown in salmon. Tachylectin 5A is colored green. The positions of bound Ca^2+^ ions bound to TNR and tachylectin 5A are indicated by grey and green spheres, respectively. The N-acetyl-D-glucosamine (labeled GlcNAc) bound in the carbohydrate-binding site of tachylectin 5A is shown in stick representation and colored purple. The letters N and C indicate the N- and C-termini, respectively.
2. Amino acid conservation at the ligand-binding site in the Fg domains of human TNR and tachylectin 5A. Identical residues are shaded in black. Green triangles below the sequence indicate residues in the carbohydrate-binding site of tachylectin 5A that do not have a structural equivalent in the RPTPζ-binding site of TNR. Salmon triangles denote residues in the RPTPζ-binding site of TNR that do not have a structural equivalent in the carbohydrate-binding site of tachylectin 5A. Purple triangles indicate residues that are found both in the RPTPζ-binding site and sugar-binding site of TNR and tachylectin, respectively. The numbering above the sequence corresponds to human TNR.
3. Amino acid residues in the interface between GlcNAc and tachylectin 5A and between RPTPζ and TNR are shown in the left and middle panels, respectively. An overlay of the two interfaces is shown in the right panel. For tachylectin 5A, the list of interacting residues was obtained from Kairies et. al (32). Residues in tachylectin 5A, RPTPζ, and TNR are coloreored green, slate, and salmon, respectively, while the bound sugar is colored purple. This analysis shoes that residues in RPTPζ-binding site in TNR(Fg) overlap with those found in the carbohydrate-binding site in tachylectin 5A.

Fg, fibrinogen; GlcNAc, N-acetyl-D-glucosamine; TNR, tenascin-R; RPTPζ, protein tyrosine phosphatase receptor type Z
